# Epithelial-mesenchymal transition and nuclear β-catenin induced by conditional intestinal disruption of *Cdh1* with *Apc* is E-cadherin EC1 domain dependent

**DOI:** 10.18632/oncotarget.11513

**Published:** 2016-08-23

**Authors:** Julia Matheson, Claudia Bühnemann, Emma J. Carter, David Barnes, Hans-Jürgen Hoppe, Jennifer Hughes, Stephen Cobbold, James Harper, Hans Morreau, Mirvat Surakhy, A. Bassim Hassan

**Affiliations:** ^1^ Tumour Growth Group, Oxford Molecular Pathology Institute, Sir William Dunn School of Pathology, University of Oxford, South Parks Road, Oxford, United Kingdom; ^2^ Department of Pathology, Leiden University Medical Centre, Leiden, The Netherlands

**Keywords:** E-cadherin, Apc, intestine, β-catenin, adhesion complex

## Abstract

Two important protein-protein interactions establish E-cadherin (*Cdh1*) in the adhesion complex; homophilic binding via the extra-cellular (EC1) domain and cytoplasmic tail binding to β-catenin. Here, we evaluate whether E-cadherin binding can inhibit β-catenin when there is loss of Adenomatous polyposis coli (APC) from the β-catenin destruction complex. Combined conditional loss of *Cdh1* and *Apc* were generated in the intestine, intestinal adenoma and adenoma organoids. Combined intestinal disruption (*Cdh1^fl/fl^Apc^fl/fl^Vil-CreERT2*) resulted in lethality, breakdown of the intestinal barrier, increased *Wnt* target gene expression and increased nuclear β-catenin localization, suggesting that E-cadherin inhibits β-catenin. Combination with an intestinal stem cell *Cre* (*Lgr5CreERT2*) resulted in *Apc^Δ/Δ^* recombination and adenoma, but intact *Cdh1^fl/fl^* alleles. Cultured *Apc^Δ/Δ^Cdh1^fl/fl^* adenoma cells infected with adenovirus-Cre induced *Cdh1^fl/fl^* recombination (*Cdh1Δ/Δ*), disruption of organoid morphology, nuclear β-catenin localization, and cells with an epithelial-mesenchymal phenotype. Complementation with adenovirus expressing wild-type *Cdh1* (Cdh1-WT) rescued adhesion and β-catenin membrane localization, yet an EC1 specific double mutant defective in homophilic adhesion (Cdh1-Mut^W2A, S78W^) did not. These data suggest that E-cadherin inhibits β-catenin in the context of disruption of the APC-destruction complex, and that this function is also EC1 domain dependent. Both binding functions of E-cadherin may be required for its tumour suppressor activity.

## INTRODUCTION

E-cadherin (*Cdh1*), a type 1 trans-membrane glycoprotein and a key structural and regulatory component of epithelial adherence junctions, links cell-cell binding to the dynamic cytoskeleton [1, 2]. It has two principal protein binding interactions. The N-terminal EC1 domain of the extra-cellular immunoglobulin like domains (EC1-EC5) binds by a homophilic interaction in *trans*, with evidence for clustering in *cis* through weaker EC2 and EC3 two-dimensional interactions [3, 4]. The EC1 strand swapping mechanism occurs via calcium dependent binding of a tryptophan (Trp2) residue to the complimentary EC1 hydrophobic pocket, in a mechanism that also involves the N-terminal 1-10 amino acids that contain a proline hinge region (residues 4-6) that facilitates Trp2 binding [3]. E-cadherin also constitutively binds β-catenin, α-catenin and p120 catenin via a cytoplasmic tail domain, to form a complex that interacts with the attached actin cytoskeletal, and post-translational signalling components that regulate cell shape and motility [5].

E-cadherin is pre-bound to β-catenin as it exits the endoplasmic reticulum [6], suggesting that the cytoplasmic and membrane tethered pools of β-catenin may be plausibly regulated by E-cadherin. E-cadherin binds to β-catenin and a type I*γ* phosphatidylinositol phosphate kinase (PIPKI*γ*) in complex, and is stabilized at the juxta-membrane by p120 catenin and binding to the F-actin cytoskeleton. This complex functions to limit the normal ubiquitin mediated degradation of E-cadherin via Hakai [7–9]. The adhesion complex also localises p120-binding proteins including PLEKHA7 and a cytoplasmic DROSHA dependent pri-miRNA processing complex [10]. When β-catenin becomes detached from E-cadherin in the adhesion complex, it is then regulated in the cytoplasm by a destruction complex. This complex comprises Adenomatous Polyposis Coli (APC), glycogen synthase kinase 3 alpha/beta (GSK3β), casein kinase 1 (CK1) and Axin1, and acts to limit the activation of β-catenin/Lef/TCF dependent gene expression [11, 12]. Here we address whether the nuclear supply of β-catenin is also inhibited by E-cadherin, especially in the cancer specific context of loss of function of the APC associated destruction complex, and whether inhibition is extra-cellular adhesion domain dependent [5].

The mouse intestinal epithelium is an ideal model to genetically evaluate mammalian E-cadherin, APC and β-catenin function, as it is comprised of multiple parallel orientated crypt-villus functional adherent cell units that derive from a *Wnt* pathway regulated crypt stem cell compartment [13]. Leucine-rich repeat containing G-protein-coupled receptor (*Lgr5*) positive stem cells, flanked by Paneth cells, have a key function in maintaining the stem cell niche through *Wnt* growth factor activation of β-catenin stability [14, 15]. *Lgr5* positive columnar stem cells divide, renew and create an adherent population of transit amplifying cells that then undergo 4 to 5 cycles of cell division before differentiating and ascending up the villus [15, 16]. Differentiated cells are comprised of four main epithelial lineages, enterocytes, goblet, entero-endocrine and Paneth cells. The latter migrate to the crypt base where they reside for 3-6 weeks, whereas the remaining cells escalate to the tip of the villi over 3-5 days prior to separation, anoikis and shedding, the latter associated with E-cadherin degradation [17, 18]. Importantly, β-catenin supply has been shown to be essential for crypt-villus homeostasis and the development of intestinal adenoma and carcinoma [19, 20].

Constitutive activation of the *Wnt* pathway is considered the driver for colorectal cancers (CRCs), most frequently due to loss of function of *APC* and disruption of the destruction complex (70-80%), but also through specific gain of function β-catenin mutations (10-15%), mutation of RNF43 (15-18%) and more rarely through a DNA translocation that induces over-expression of R-Spondin (<5%) [21–23]. In the absence of Wnt and R-Spondin activation of a receptor complex comprised of Lgr5/Frizzled/Lrp6/ZNRF3, β-catenin is phosphorylated, ubiquitinated and degraded within the destruction complex, so precluding its nuclear localization with Lef1/Tcf4 transcription factors [12]. *APC* truncation mutations and loss of heterozygosity, can result in complete loss of β-catenin destruction, leading to increased Tcf/β-catenin downstream target gene expression, e.g. *Myc*, *Axin2* and *CD44*, effects that are reversible following *Apc* re-expression [19, 20, 24]. Recently, evidence in mouse intestinal adenoma models suggest that E-cadherin is able to reduce the supply of stabilised β-catenin associated with a somatic gain of function mutation [25]. Moreover, genome wide association studies in colorectal cancer have identified SNP (rs9929218) of *CDH1* as a germ-line modifier of colorectal cancer susceptibility and survival, yet the mechanistic basis of this association, and the potential interaction with multiple subsequent somatic acquired mutations, remains unknown [26, 27].

To evaluate the combined functional effects of *Cdh1* and *Apc* in the intestine, we utilised conditional genetic models. Conditional alleles (loxP) of genes *Cdh1* and *Apc* were combined using breeding, and recombination induced by additional expression of intestinal specific Cre transgenes. Here, we denote the germ-line determined detection of homozygote loxP alleles as *Cdh1^fl/fl^* and *Apc^fl/fl^*. Where somatic tissues have been directly tested for the presence of the resulting Cre induced recombined alleles using PCR, we denote the alleles as *Cdh1^Δ/Δ^* and *Apc^Δ/Δ^*. Adenoma organoid cultures derived directly from adenoma tissue *in vivo,* where alleles were recombined (e.g. PCR positive for *Apc^Δ/Δ^*), were then genetically complemented with *Cdh1* expressed using adenoviral expression vectors, and phenotypes evaluated. Our findings suggest significant E-cadherin inhibition of β-catenin supply in the context of *Apc* genetic disruption. These data have functional mechanistic implications for invasive human cancers with co-existing *APC* and *CDH1* loss of function.

## RESULTS

### Combined intestinal Apc and Cdh1 loss of function show additive phenotypes

The *Vil-Cre* transgene is expressed from E9 in the visceral endoderm of the yolk sac and by E12.5 in the developing intestinal epithelium [28]. Assessment of genotypes from breeding of *Cdh1^+/fl^Vil-Cre* × *Cdh1^+/fl^Vil-Cre,* or *Cdh1^fl/fl^Vil-Cre* × *Cdh1^+/fl^Vil-Cre* animals showed a highly significant deviation from the expected Mendelian ratios at birth (P0, see Supplementary Figure S1a). Expected ratios and normal morphology of embryos were observed at E10.5, but by E11.5-E12.5 there were increased numbers of embryonic resorptions (Supplementary Figure S1b). Following Cre-mediated recombination, *Cdh1^Δ/Δ^Vil-Cre* embryos appeared smaller than littermate controls, and frequently lacked extra-embryonic membranes, and by E12.5, had reduced yolk sac E-cadherin labelling (Supplementary Figure S1c) [29].

**Figure 1:**
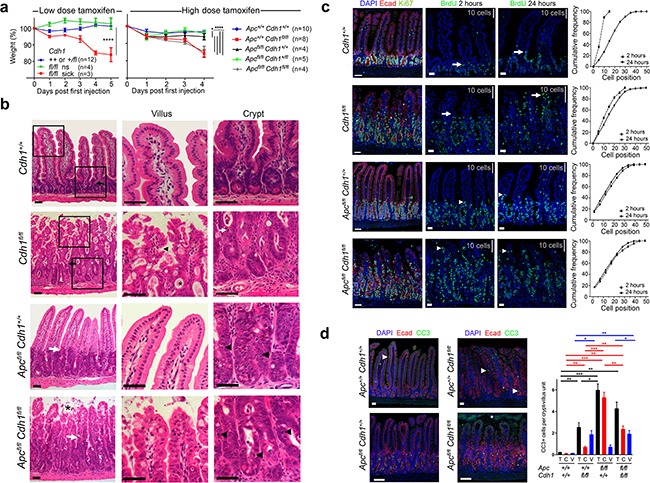
Intestinal phenotype following conditional *Cdh1* and *Apc* homozygous disruption with *Vil-CreER^T2^* **a.** Animal weight (% of pre-injection) after intra-peritoneal injection with either low (40 mg.kg^−1^ d1-5) or high dose tamoxifen (200 mg.kg^−1^ d1-2), to generate *Cdh1^Δ/Δ^* with and without *Apc^Δ/Δ^*. Animals became either sick or not-sick (ns). ANOVA with Bonferroni post test for each genotype (±SEM). * p<0.05. **** p<0.001. **b.** H and E staining of small intestine, with high power views of villus and crypt. Black and white arrows locate the crypt-villus boundary under low power. In *Cdh1^Δ/Δ^*, black arrowhead villus tufting, *mucus pooling, white arrow apoptotic nuclei. In *Apc^Δ/Δ^Cdh1^Δ/Δ^*, * villous shedding, black arrow heads indicate more frequent apoptotic nuclei. **c.** Intestinal proliferation detected with BrdU labelling following injection either 2 or 24 hours prior to dissection, compared to Ki-67. White arrows/arrowheads indicate the leading edge of BrdU labelling, grey bars indicate distance spanned by 10 crypt-villous cells. Cumulative frequency plots quantifying BrdU labelling, with cell position on the crypt-villus axis from the crypt base. **d.** Labelling for cleaved caspase 3 (CC3), number of positive cells per crypt-villus unit, *=shed cells in the intestinal lumen that are CC3 positive but not counted, white arrowhead positive labelling. Quantification of CC3 positive cells, total (black), crypt (red) and villi (blue), ≥3 animals per genotype and ≥20 crypt-villus units analysed per animal, ±SEM. Student's t-test, * p<0.05, ** p<0.01, *** p<0.005. Image bars 50 μm.

**Figure 2:**
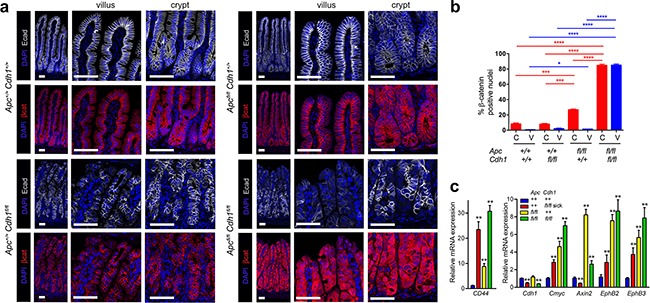
Combined *Vil-CreER^T2^* with *Apc* and *Cdh1* homozygous floxed alleles results in β-catenin nuclear localization and activation of *Wnt* target genes **a.** Localization of E-cadherin and β-catenin in the small intestine from *Apc^+/+^Cdh1^+/+^Vil-CreER^T2^*, *Apc^+/+^Cdh1^fl/fl^Vil-CreER^T2^*, *Apc^fl/fl^Cdh1^+/+^Vil-CreER^T2^* and *Apc^fl/fl^Cdh1^fl/f^, Vil-CreER^T2^*. Loss of E-cadherin appears patchy presumably because of intestinal regeneration. *Apc^Δ/Δ^Cdh1^Δ/Δ^Vil-CreER^T2^* is associated with marked cytoplasmic and nuclear localization of β-catenin. Scale bars 50μm. **b.** Quantification of nuclear β-catenin in crypt or villus from each genotype (n=3 animals per genotype, >1000cells counted per genotype (±SEM). Student t-test. * p<0.05, ** p<0.01, *** p<0.005, ****p<0.001. (c) RT-QPCR of whole intestinal samples for *Wnt* target genes. (n≥3 mice per genotype) relative to *Actb* and *Hprt* expression and wild-type small intestine. *Apc* and *Cdh1* homozygous floxed intestinal cells over-express *Wnt* target genes. Student's t-test (±SEM) **p<0.01.

To sidestep embryonic lethality, a tamoxifen inducible intestinal *Vil-CreER^T2^* transgene was then combined with *Cdh1* loxp alleles [28]. Following induction with 40 mg.kg^−1^ tamoxifen administered by intra-peritoneal injection on five sequential days (low dose tamoxifen) or 200 mg.kg^−1^ on two sequential days (high dose tamoxifen), adult animals either remained well (*Cdh1^+/+^Vil-CreER^T2^*, *Cdh1^+/fl^Vil-CreER^T2^*) or became sick (*Cdh1^fl/fl^Vil-CreER^T2^*) after 4-5 days, with weight loss, dehydration and diarrhoea (Figure 1a). At post-mortem, *Cdh1^fl/fl^Vil-CreER^T2^* animals had developed peritoneal exudates, grossly dilated intestines that were also significantly shortened and hyperaemic (Supplementary Figure S2a,b). Genotyping of the *Cdh1* allele, labelling for YFP and E-cadherin, and RT-QPCR, all confirmed efficient intestinal recombination (*Cdh1 ^Δ/Δ^*) with <50% *Cdh1* mRNA expression (Supplementary Figure S2c). *Cdh1^Δ/Δ^Vil-CreER^T2^* small intestines had distorted crypt-villus architecture, loss of the well-defined crypt-villus boundary, loss of uniform cell stacking along the crypt-villus axis with mucus pooling, villus tufting, epithelial cell shedding and inflammatory infiltrates (Figure 1b). RT-QPCR of spleen material for bacterial ribosomal S16 RNA (a component of the 30S small subunit of prokaryotic ribosomes) confirmed *Cdh1^fl/fl^Vil-CreER^T2^* animals had evidence of systemic bacteraemia secondary to breakdown of the intestinal barrier (Supplementary Figure S2d). Evidence of intestinal regeneration in *Cdh1^fl/fl^Vil-CreER^T2^* animals was supported by an increase in crypt cellular proliferation *Cdh1^fl/fl^Vil-CreER^T2^* labeled with BrdU, and confirmed by Ki-67 immuno-labelling (Figure 1c). Apoptosis assessed by cleaved caspase-3 labelling was also significantly increased in *Cdh1^Δ/Δ^Vil-CreER^T2^* villi (Figure 1d). Significant increases in the Wnt target genes, *c-Myc*, *CD44* and *EphB3* were detected, but with decrease of *Axin2* mRNA (Figure 2c). *Axin2* is generally considered to be a reliable Wnt pathway reporter (http://www.stanford.edu/group/nusselab/cgi-bin/wnt/main), and is used in preference to transgenic *Wnt* reporters [30]. Paneth cells were scattered along the crypt-villus axis (Supplementary Figure S3a,b), goblet cells (Mucin 2) and enteroendocrine cells (Chromogranin A) were also significantly reduced in number and predominantly located in the upper villus compared with wild-type animals (Supplementary Figure S3a,b). Of the surviving cohort of *Cdh1^fl/fl^Vil-CreER^T2^* treated with low dose tamoxifen and aged to one year, neither adenoma nor tumors formed (*Cdh1^fl/fl^Vil-CreER^T2^* n=4, *Cdh1^+/fl^Vil-CreER^T2^* n=6, *Cdh1^+/+^Vil-CreER^T2^* n=3).

We next investigated the consequence of *Apc* loss of function combined with *Cdh1*. Intestinal activation of *Vil-CreER^T2^* in *Apc^fl/fl^* resulted in a phenotype similar to that previously characterized using *AhCreER^T^* and *Vil-CreER^T2^* [24]. *Apc^fl/fl^Vil-CreER^T2^* animals became unwell with weight loss and diarrhoea five days following tamoxifen treatment (Figure 1a). Unlike *Cdh1^fl/fl^Vil-CreER^T2^*, there was no evidence of intestinal shortening or inflammation (Supplementary Figure S2a,b). For *Apc^fl/fl^Vil-CreER^T2^* intestine, expanded crypt zones with densely stacked cells, increased apoptotic nuclei, and a clear boundary between the proliferative zone and the villus, were observed (Figure 1b,c). When *Apc^fl/fl^* and *Cdh1^fl/fl^* were then combined with *Vil-CreER^T2^*, rapid phenotypic changes with weight loss, diarrhoea, intestinal dilatation and shortening, all occurred within 5 days (Supplementary Figure S2). Histology of the intestines showed combined phenotypic features attributable to both *Apc^Δ/Δ^* and *Cdh1^Δ/Δ^*, *e.g.* an expanded crypt zone, disorganized crypt structure and crypt-villus boundary, and shedding of cells from the villus tip (Figure 1b). *Apc^Δ/Δ^Cdh1^Δ/Δ^Vil-CreER^T2^* small intestines labelled with an E-cadherin antibody showed areas of loss that were particularly marked in the villus region, with patchy loss in the expanded crypt zone and retained labelling in some cells (Figure 1c,d). In *Apc^Δ/Δ^Vil-CreER^T2^* intestines, the crypt-villus boundary was labelled by c-Myc and EphB2, but in *Apc*^Δ/Δ^*Cdh1*^Δ/Δ^*Vil-CreER^T2^* intestines this had a less defined border (Supplementary Figure S4a,b,c). As with *Apc*^Δ/Δ^*Vil-CreER^T2^* intestines, there was significantly increased proliferation and apoptosis in the expanded crypt zone, but this also extended into the villi as in *Cdh1^fl/fl^Vil-CreER^T2^* (Figure 1d). The most striking feature appeared the localization of β-catenin, where marked increases in cytoplasmic and nuclear β-catenin were observed in the *Apc*^Δ/Δ^*Cdh1*^Δ/Δ^*Vil-CreER^T2^* intestinal crypts and villi (Figure 2a,b). Nuclear β-catenin was quantified and was present in a significantly higher percentage of cells in both the crypt and the villi (Figure 2b). RT-QPCR showed expected up-regulation of *Wnt* target genes including *c-Myc and CD44* (p=0.02 and 0.0007 respectively, Figure 2c), although Axin 2 appeared to have increased to a lesser extent when comparing *Apc*^Δ/Δ^*Cdh1*^Δ/Δ^*Vil-CreER^T2^* to *Apc*^Δ/Δ^*Vil-CreER^T2^* intestinal controls (p=0.007, Figure 2c). The increase in mRNA was confirmed by labelling for CD44, c-Myc, EphB2 and EphB3 (Supplementary Figure S4 a,b,c). Overall, these data suggest that E-cadherin inhibits nuclear β-catenin localization when there is Apc loss of function, and that this correlates with gene expression, although the effect on *Axin2* appeared different and unexpected (Figure 2c and Supplementary Figure S5).

Lysozyme labelled Paneth cells were re-positioned away from the crypt base but were still confined to the expanded proliferative zone, whereas goblet and entero-endocrine cell numbers appeared decreased in *Apc*^Δ/Δ^*Cdh1*^Δ/Δ^*Vil-CreER^T2^* intestines compared to *Apc*^Δ/Δ^*Vil-CreER^T2^* controls (Supplementary Figure S3a,b). In *Apc*^Δ/Δ^*Cdh1*^Δ/Δ^*Vil-CreER^T2^* intestines, cells appeared less densely packed in the expanded proliferative zone with lower total number of cells along the crypt-villus axis (Figure 1c). The proliferative zone was expanded throughout the crypts and villi as a result of *Apc*^Δ/Δ^** and *Cdh1*^Δ/Δ^**indicated by BrdU labelling (Figure 1c). Apoptosis was also markedly increased in the expanded proliferative zone of *Apc*^Δ/Δ^*Cdh1*^Δ/Δ^*Vil-CreER^T2^* intestines, with non-adherent cells displaying high expression of cleaved caspase 3 cells in the intestinal lumen (Figure 1d).

Gene Set Enrichment Analysis (GSEA) was performed on intestinal RNA microarray data derived from all genotypes and using all of the gene sets in the Molecular Signatures Database v4.0 (Broad Institute). We identified the leading edge sets (LES) from this analysis, containing 955 genes, including those associated with Wnt pathway activation, and interrogated these data further by extracting the genes which occurred most frequently in LES (Supplementary Figure S5). Aside from confirming Wnt pathway genes, the most enriched was *Areg* (Amphiregulin), coding for an EGF receptor ligand, upregulated in *Apc*^Δ/Δ^*Cdh1*^Δ/Δ^*Vil-CreER^T2^* intestines. We also selected genes that are present in the most recent Sanger COSMIC Cancer Genes census (http://cancer.sanger.ac.uk/cancergenome/projects/census/), and those with a Gene Ontology (GO) term annotated as ‘inflammatory’. A total of 5 genes were annotated for both cancer and inflammation: *Fcgr2b*, *Egfr*, *Tnfaip3*, *Fas* and *Myd88*.

### Combined Apc and Cdh1 recombination induced intestinal adenoma

To further investigate the genetic interactions between *Apc* and *Cdh1* in the intestine, we next combined genotypes in intestinal adenoma. Heterozygote alleles were combined (*Apc^fl/+^Cdh1^fl^*^/+^*Vil-CreER^T2^*) and intestinal adenoma induced by tamoxifen and results compared to that of germ-line heterozygotes *Cdh1^+/−^* combined with the *Apc^Min/+^* model (Supplementary Figure S6). Our initial results suggested that *Cdh1* heterozygosity might promote *Apc* loss of function induced adenoma formation. We observed, however, a strain dependent effect of *Cdh1* with both *Apc^Min/+^* (129Ola) and *Apc^fl/+^*(B6D2)*Vil-CreER^T2^,* as the differences in adenoma counts and survival between either *Apc^Min/+^* or *Apc^fl/+^* diminished when the alleles were back-crossed from a mixed background to C57BL/6J, consistent with the presence of non- C57BL/6J modifier alleles [31]. Moreover, these data suggest the decreased survival and greater adenoma frequency previously reported with *Cdh1* heterozygosity may be because of co-segregating modifiers from a mixed strain background, as well as being potentially *Cdh1* dependent [32].

**Figure 3:**
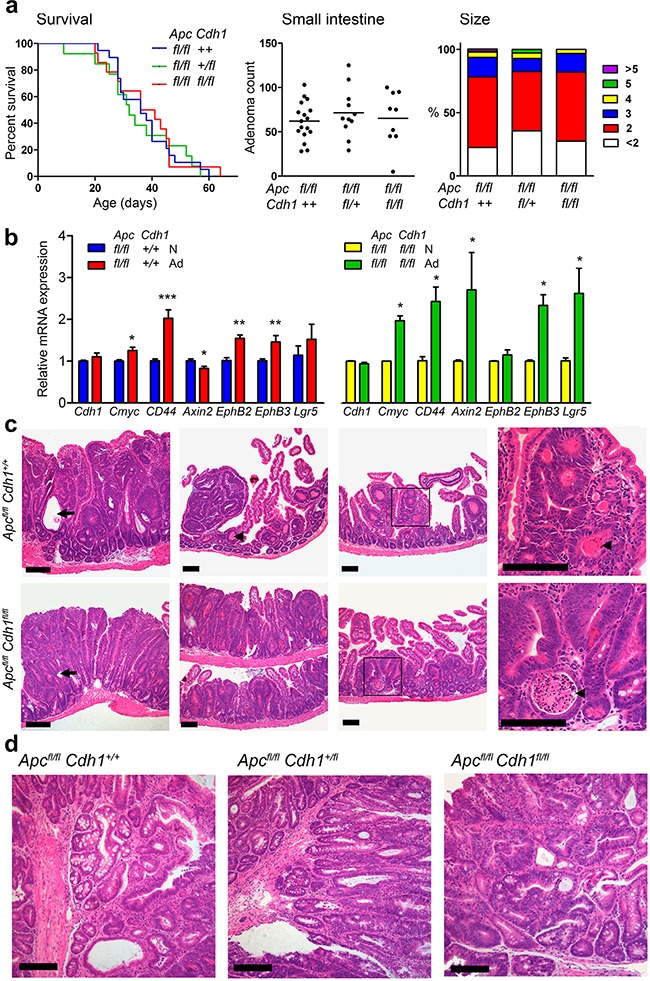
*Lgr5CreER^T2^* induces *Apc^fl/fl^* floxed intestinal adenoma without co-floxed *Cdh1* alleles **a.** Survival analysis following combination of *Apc^fl/fl^Cdh1^fl/fl^* with *Lgr5CreER^T2^*. Induced *Apc^Δ/Δ^* adenoma reduced survival. No significant differences were observed between genotypes (log rank test, *Apc^fl/fl^Cdh1^+/+^* n=19, *Apc^fl/fl^Cdh1^fl/+^* n=13, *Apc^fl/fl^Cdh1^fl/fl^* n=14). Small intestine adenoma counts and adenoma diameters (mm) in mice sacrificed at the humane endpoint, showing no significant difference between genotypes. One-way ANOVA with Bonferroni post-test. (*Apc^fl/fl^Cdh1^+/+^* n=17, *Apc^fl/fl^Cdh1^fl/+^* n=11, *Apc^fl/fl^Cdh1^fl/fl^* n=9). **b.** RT-QPCR of *Cdh1* and *Wnt* target genes in caecal adenoma (Ad) compared with normal caecal tissue (N). Up-regulation of relative expression of *Wnt* target genes, normalized with *β-actin* and *Hprt* (Normal intestine control gene expression not significantly different; *β-actin CT*=16.0±0.58 *vs* 16.5±0.76*, Hprt CT=*23.9±0.51 *vs* 24.0±0.53 *for Apc^fl/fl^Cdh1^+/+^*[n=8] and *Apc^fl/fl^Cdh1^fl/fl^*[n=5], respectively), in adenomas genotypes. Note *Cdh1* mRNA expression persists indicating lack of *Cdh1* floxed alleles. n>3 animals. Student's t-test (±SEM). *p<0.05. **p<0.01. ***p<0.001. **c.** Representative histology (H&E) of small intestinal adenoma from tamoxifen treated *Lgr5-CreER^T2^* on *Apc^fl/fl^*, *Cdh1^+/+^* and *Apc^fl/fl^*, *Cdh1^fl/fl^* backgrounds showing sessile small intestinal adenomas, diffuse adenomatosis, necrosis(arrowheads, necrotic cellular debris within the lumen of neoplastic glands) and cystic changes (arrows) in both genotypes. **d.** Representative histology (H&E) of caecal tumours from the same animals showing a similarspectrum of adenoma grades. Scale bars 100μm. See Supplementary Figure S7 for Tamoxifen re-challenge.

We next combined *Apc^fl/fl^Cdh1^fl/fl^* and *Apc^fl/fl^Cdh1^+/+^* with a *Cre* driven by the promoter of the *Wnt* response gene and stem cell marker *Lgr5*, (Lgr5-EGFP-IRES-CreER^T2^ or *Lgr5CreER^T2^*) [14, 33]. Recombination rates in intestinal epithelial cells are lower overall with this transgene (5-6%), but highly specific for *Lgr5* stem cells. Rapid adenoma development within 3-5 weeks occurs as a result of bi-allelic *Apc*^Δ/Δ^** recombination. Surprisingly, comparison of overall survival and adenoma formation between *Apc^fl/fl^Cdh1^fl/fl^Lgr5CreER^T2^*, *Apc^fl/fl^Cdh1^+/fl^Lgr5CreER^T2^* and *Apc^fl/fl^Cdh1^+/+^Lgr5CreER^T2^* showed no significant differences (Figure 3a). Wnt dependent gene expression in adenoma was markedly increased as observed in the small intestine (Figure 3b). Histological grading of small intestinal adenoma showed low-grade dysplasia in all genotypes, with caecal tumours rarely exhibiting high-grade dysplasia and carcinoma *in situ* (Figure 3c,d). Genotyping of adenoma showed most retained *Cdh1* non-recombined (*Cdh1^fl/fl^*) alleles, with a minority of adenoma showing evidence of recombination by PCR, although similar detectable *Cdh1* mRNA expression was seen irrespective of genotype (Figure 3b, Supplementary Figure S7c). E-cadherin labelling of adenoma revealed some areas of reduced E-cadherin, but no areas of complete loss within adenoma from *Apc^fl/fl^Cdh1^fl/fl^Lgr5CreER^T2^* genotyped mice (Supplementary Figure S7d). These data suggest that there was either inefficient recombination, or frequent recombination but negative selection of double homozygote recombined cells (*Apc*^Δ/Δ^*Cdh1*^Δ/Δ^**).

In order to test whether recombination could be further induced, we re-challenged animals with *Apc*^Δ/Δ^*Cdh1^fl/fl^Lgr5CreER^T2^* adenoma with a second series of tamoxifen injections, in the expectation that as *Lgr5* expression is also activated by the Wnt pathway, repeated recombination would increase the detection of *Cdh1*^Δ/Δ^** within adenoma, [14, 34]. Three animals re-injected at 16 days after the first round of tamoxifen injections, showed an expected increase in small sized adenoma as a result of a further wave of *Apc^fl/fl^* recombination (*Apc*^Δ/Δ^**). All three animals deteriorated rapidly however, two within 4 days of tamoxifen re-challenge, yet all had a similar adenoma counts (Supplementary Figure S7a). The increase in the number of smaller adenoma after re-challenge was expected, as recombination of *Apc^fl/fl^* would increase the number of adenoma initiated, but this effect was unlikely to account for the rapid deterioration of the animals (Supplementary Figure S7a). Necropsy revealed evidence of peritonitis, suggesting intestinal perforation, with histological examination showing areas of blood, necrosis and cell death within adenoma consistent with the potential for a localised *Cdh1*^Δ/Δ^** induced loss of the intestinal barrier within adenoma (Supplementary Figure S7b). Overall, these data suggest that *Apc*^Δ/Δ^*Cdh1*^Δ/Δ^** adenoma cells could be generated within existing adenoma, but that these cells had either impaired survival or were rapidly removed, as they were not frequently detected.

### Conditional Cdh1 disruption in Apc induced adenoma cells following in vitro organoid culture

**Figure 4:**
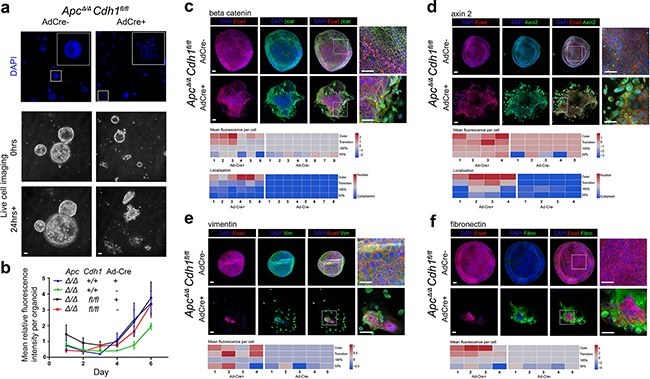
Adenovirus-Cre expression in *Apc^Δ/Δ^Cdh1^fl/fl^* intestinal adenoma organoids induces floxed *Cdh1^Δ/Δ^* alleles, adenoma disruption and a mesenchymal phenotype **a.** Ad-Cre-GFP treatment of *Apc^Δ/Δ^Cdh1^fl/fl^* adenoma organoids, labeled with DAPI, and frames from live cell imaging (baseline and after 24 hours; see Supplementary movies). *Apc^Δ/Δ^Cdh1^fl/fl^*Ad-Cre-GFP organoids show a fragmented appearance at low power, four days following Ad-Cre-GFP transfection and culture. Scale bars 100μm. **b.** Growth of adenoma organoids quantified with total DAPI fluorescence (>10 intact adenoma per time-point). No significant genotype differences in organoid growth were observed. **c-f.** Labeling of *Apc^Δ/Δ^Cdh1^fl/fl^* adenoma regions and cellular localization following Ad-Cre-GFP transfection for (c) β-catenin, (d) axin2, (e) vimentin and (f) fibronectin, displayed as heatmaps (fluorescence signal log_2_ transformed, see Methods and Supplementary Figure S12). Note outer organoid localization of vimentin and fibronectin, with β-catenin and axin2 nuclear labeling. Scale bars 50μm.

**Figure 5:**
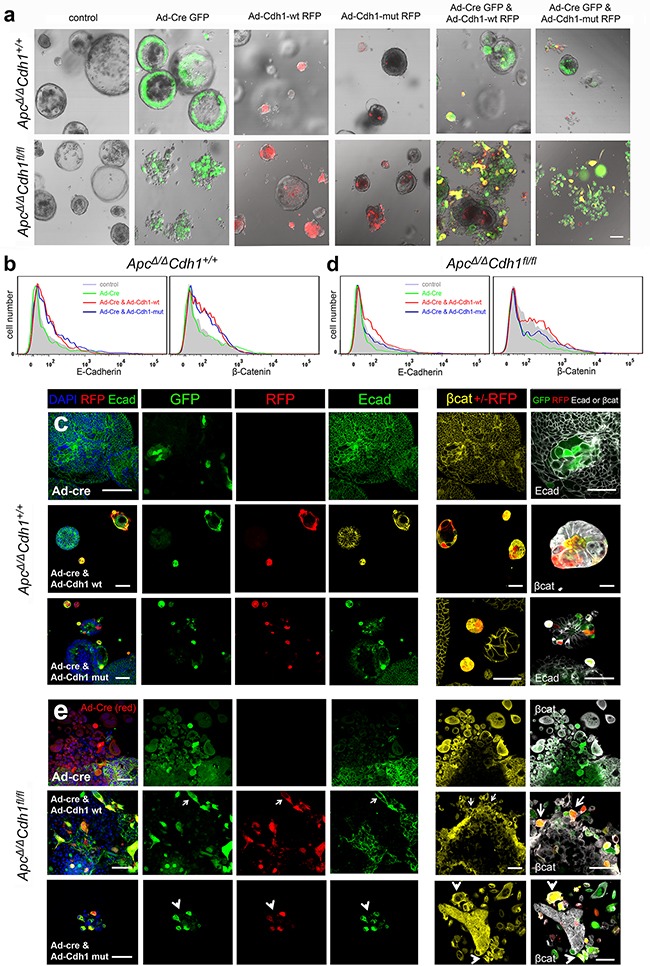
Genetic complementation of *Apc^Δ/Δ^Cdh1^fl/fl^* adenoma organoid cells with adenovirus either expressing wild-type or EC1 domain mutants of *Cdh1* **a.** Adenovirus transfection of *Apc^Δ/Δ^Cdh1^+/+^*and *Apc^Δ/Δ^Cdh1^fl/fl^* intestinal adenoma organoids with Ad-Cre-GFP, Ad-Cdh1-WT-RFP and Ad-Cdh1-Mut-RFP. Ad-Cre-GFP is expressed in the outer cells of the *Apc^Δ/Δ^Cdh1^+/+^* control organoid, and in cells of the disrupted organoid in *Apc^Δ/Δ^Cdh1^fl/fl^*. Expression of either Ad-Cdh1-WT-RFP or Ad-Cdh1-Mut-RFP results in small contracted organoids in both genotypes. Complementation of *Apc^Δ/Δ^Cdh1^fl/fl^*Ad-Cre-GFP with Ad-Cdh1-WT-RFP, results in adherent double expressing (yellow) cells in both genotypes. For complementation with Ad-Cdh1-Mut-RFP, double expressing cells (yellow) appear less adherent and separate. **b** & **d.** Flow cytometry profiles of E-cadherin and β-catenin antibody binding to *Apc^Δ/Δ^Cdh1^+/+^* adenoma organoid cells with adenoviral transfection. With Ad-Cre-GFP alone, there was no effect on overall E-cadherin and β-catenin expression, but when combined with either Ad-Cdh1-WT-RFP or Ad-Cdh1-Mut-RFP, results in increased E-cadherin and β-catenin. In *Apc^Δ/Δ^Cdh1^fl/fl^* cells, loss of E-cadherin and β-catenin occurs following Ad-Cre-GFP transfection, and is rescued by either Ad-Cdh1-WT-RFP or Ad-Cdh1-Mut-RFP. (c) In *Apc^Δ/Δ^Cdh1^+/+^*, infection with Ad-Cre-GFP does not result in E-cadherin loss (top row), and Ad-Cdh1-WT-RFP (middle row) or Ad-Cdh1-Mut-RFP (bottom row) expression results in contracted organoids as in a. Note that β-catenin appears cytoplasmic and membrane bound. **e.** In *Apc^Δ/Δ^Cdh1^fl/fl^* (top row) Ad-Cre-GFP transfection results in loss of E-cadherin, cytoplasmic and nuclear localisation of β-catenin, and disrupted organoid morphology (large, separate, rounded or elongated) as in a. Complementation of *Apc^Δ/Δ^Cdh1^fl/fl^* is shown in two example panels. For Ad-Cre-GFP with Ad-Cdh1-WT-RFP (middle row), results in adherent double expressing (yellow) cells, associated with expression of E-cadherin (arrows, left panel) and predominantly membrane bound β-catenin (arrows, right panel). For complementation with Ad-Cdh1-Mut-RFP (yellow cells, bottom row, left panel), this results predominantly in non-adherent cells, with mainly cytoplasmic and nuclear β-catenin expression (white arrow heads, right panel). Scale bar 100μm in (a), otherwise 50μm (c, e).

In view of the lack of detectable *Cdh1^fl/fl^* recombination *in vivo*, we isolated *Apc*^Δ/Δ^*Cdh1^fl/fl^* adenoma cells from the small intestine, and generated adenoma suspensions for *in vitro* organoid culture [15, 35, 36]. Exposure to tamoxifen to reactivate *Lgr5CreER^T2^* in culture failed to result in recombined *Cdh1*^Δ/Δ^** alleles, confirmed by E-cadherin labelling and PCR genotyping. In order to express higher levels of *Cre*, transfection of adenoma cell suspensions was performed with an adenovirus-Cre, here with *Cre* and *GFP* expressed using a CMV promoter (Ad-Cre-GFP). Following transfection, we detected bi-allelic *Cdh1* recombined alleles (*Cdh1*^Δ/Δ^**), and a marked phenotypic change in organoid structure, with fragmentation and appearance of cells remote from the periphery of the organoids (Figure 4a, Supplementary Figure S8). Most *Apc*^Δ/Δ^*Cdh1^fl/fl^Lgr5CreER^T2^* Ad-Cre-GFP positive cells expressed high levels of Cre when labelled with an anti-Cre antibody, with significant associated loss of E-cadherin labelling (Supplementary Figure S8). Within 3 days, many Ad-Cre-GFP transfected *Apc*^Δ/Δ^*Cdh1^fl/fl^Lgr5CreER^T2^* adenoma organoids lost the spherical morula-like structure and developed surrounding regions with spindle like cell morphology (Figure 4a-f, Figure 5a). Despite the disruption of some of the *Apc*^Δ/Δ^*Cdh1^fl/fl^Lgr5CreER^T2^* organoids, we confirmed that the remaining intact *Apc*^Δ/Δ^*Cdh1^fl/fl^Lgr5CreER^T2^* and *Apc*^Δ/Δ^*Cdh^+/+^Lgr5CreER^T2^* organoids grew at similar rates (Figure 4b). Cell dispersal and cell motility were confirmed by live cell imaging (Supplementary Movies: *Apc*^Δ/Δ^*Cdh1^+/+^Lgr5CreER^T2^*Adv-Cre positive (Control Supplementary Movies 1. and 2.), *Apc*^Δ/Δ^*Cdh1^fl/fl^ Lgr5CreER^T2^*Adv-Cre positive (experimental Supplementary Movie 3.), Figure 4a, Supplementary Figure S8). After 6 days, >85% of the *Apc*^Δ/Δ^*Cdh1^fl/fl^Lgr5CreER^T2^*Ad-Cre-GFP transfected structures displayed disrupted organoid structure with separated cells, compared to <10% in *Apc*^Δ/Δ^*Cdh1^fl/fl^Lgr5CreER^T2^* non-transfected controls. Proliferation and apoptosis were quantified using EdU incorporation and cleaved caspase3 labelling, respectively. Reduced overall labelling of both markers were detected by 4-6 days after Ad-Cre-GFP transfection, although we cannot exclude apoptosis at earlier time-points (Supplementary Figure S9a,b). In cells with a ROSA26-LSL-YFP reporter incorporated into the *Apc*^Δ/Δ^*Cdh1^fl/fl^Lgr5CreER^T2^* line and transfected with Ad-Cre-GFP, an anti-GFP antibody labelled all cells. This suggested that the derived cells with associated mesenchymal phenotype and loss of E-cadherin were derived from intestinal epithelial cells (YFP or Ad*-*Cre-GFP positive, Supplementary Figure S10). As a crude control for loss of calcium mediated adhesion, EDTA exposure and culture in calcium free medium resulted in significantly different genotype independent phenotypes, with adenoma collapse, appearance of pyknotic nuclei within minutes to hours after calcium chelation, persistant labelling with CC3 and β-catenin and organoid fragmentation (Supplementary movie EDTA, Supplementary Figure S11). Importantly, no cells with a mesenchymal phenotype were observed following EDTA and low calcium media exposure.

As the disruption of adenoma organoid was mainly confined to the outer regions, we mapped the distribution of DAPI labelled cells into zones of *Apc*^Δ/Δ^*Cdh1^fl/fl^Lgr5CreER^T2^*Ad-Cre-GFP organoids, and generated heat maps reporting localization of labelling (Figure 4c-f, Supplementary Figure S12). Outer zone cells with spindle morphology had increased labelling of nuclear β-catenin and also axin 2, with cytoplasmic labelling of mesenchymal markers, fibronectin, vimentin and twist, consistent with β-catenin driven expression. As loss of E-cadherin labelling in *Apc*^Δ/Δ^*Cdh1^fl/fl^Lgr5CreER^T2^*Ad-Cre-GFP cells was associated with the emergence of a cells with a high β-catenin labelling, these data supported the function of E-cadherin to decrease β-catenin. Moreover, the phenotypic transformation of epithelial cells to a mesenchymal morphology (EMT) was also associated with increased β-catenin nuclear localization.

### Cdh1 genetic complementation of intestinal adenoma in vitro

In order to confirm that the phenotypes observed were directly attributable to *Cdh1* loss of function, we performed genetic complementation. Co-infection of *Apc^Δ/Δ^Cdh1^fl/fl^Lgr5CreER^T2^* adenoma cells with a CMV promoter driven adenovirus expressing either murine wild-type or an EC1 domain mutated *Cdh1* cDNA, were combined in vectors with an IRES for the fluorescent reporter RFP (Ad-Cdh1-WT-RFP or Ad-Cdh1-Mut-RFP). The two mutations introduced into the EC1 domain were a tryptophan to alanine (W2A), involved in the strand swapping mechanism, and a mutation of the Ca^2+^ dependent binding site that introduces a bulky tryptophan into the binding site where there is normally a serine residue (S78W). We first confirmed that these mutations led to complete loss of homophilic binding by expressing Ad-Cdh1-WT-RFP and Ad-Cdh1-Mut-RFP constructs in MCF7 cell controls (Supplementary Figure S13). Here, we utilised a murine E-cadherin-Fc domain protein to probe extra-cellular EC mediated binding function in a Ca^2+^ dependent manner using flow cytometry, and confirmed gain and loss of binding with Ad-Cdh1-WT-RFP and Ad-Cdh1-Mut-RFP, respectively. By co-infecting *Apc^Δ/Δ^Cdh1^fl/fl^Lgr5CreER^T2^* adenoma cells with Ad-Cre-GFP combined with either Ad-Cdh1-WT-RFP or Ad-Cdh1-Mut-RFP, we tested the effects of double transfection with *Cre* and *Cdh1* expressing viruses. All single virus infection MOIs resulted in >50% of cells expressing either GFP or RFP by 5-7 days, and both fluorescent reporters were detected following co-infection in proportionate expression ratios confirmed by imaging and flow cytometry (Supplementary Figure S13, Figure 5a,b).

Adenoviral transfection of *Apc^Δ/Δ^Cdh1^+/+^Lgr5CreER^T2^* control organoids with Ad-Cre-GFP did not significantly change either the expression level of E-Cadherin or morphology, as expected (Figure 5a,b). Expression of Ad-Cdh1-WT-RFP and Ad-Cdh1-Mut-RFP did however increase overall E-cadherin labelling by flow cytometry, here using an anti-E-cadherin antibody to a C-terminal epitope, with frequent appearance of much smaller adenoma compared to adenoma that were not-infected (RFP negative) within the same culture (Figure 5a,c). In *Apc^Δ/Δ^Cdh1^fl/f^Lgr5CreER^T2^* with Ad-Cre-GFP, decreased labelling for E-cadherin was observed as expected, and this was rescued by Ad-Cdh1-WT-RFP and Ad-Cdh1-Mut-RFP as judged by single channel flow cytometry (Figure 5b). On transfection of Ad-Cre in *Apc^Δ/Δ^Cdh1^fl/fl^Lgr5CreER^T2^* adenoma cells, loss of E-cadherin labelling was observed as before, and predominantly at the edge of adenomas, where separated rounded and elongated cells were visible (Figure 5a). Fluorescent labelling of cells and localisation using confocal imaging revealed an increase in overall β-catenin labelling in both the nucleus and cytoplasm in cells that had lost E-cadherin, and again with larger, separated, rounded and elongated cells (Figure 5e, top row). RFP labelled cells showed increased E-cadherin expression following either Cdh1-WT-RFP or Cdh1-Mut-RFP transfection as judged by flow cytometry, recovering overall levels to those of wild-type (Figure 5b). The morphology of the *Apc^Δ/Δ^Cdh1^+/+^Lgr5CreER^T2^* compared to the *Apc^Δ/Δ^Cdh1^fl/fl^Lgr5CreER^T2^* cells complemented with either Cdh1-WT-RFP or Cdh1-Mut-RFP, also differed. For *Apc^Δ/Δ^Cdh1^+/+^Lgr5CreER^T2^,* adherent and smaller cells occurred in Ad-Cre-GFP and Cdh1-WT-RFP double-labelled cells (yellow) (Figure 5c). In contrast, generally isolated and non-adherent cells occurred in Ad-Cre-GFP and Cdh1-Mut-RFP double-labelled cells (yellow), (Figure 5a,e bottom row). Re-expression of Cdh1-WT-RFP in Ad-Cre-GFP transfected *Apc^Δ/Δ^Cdh1^fl/fl^Lgr5CreER^T2^* cells appeared to result in expression of E-cadherin (Figure 5e middle row, left panel), clear membrane-bound β-catenin labelling, and adhesive and less rounded and flattened morphology (Figure 5e, middle row, right panel, arrows), For Cdh1-Mut-RFP co-infected *Apc^Δ/Δ^Cdh1^fl/fl^Lgr5CreER^T2^* cells, β-catenin labelling remained high and appeared cytoplasmic and nuclear with cells appearing rounded and non-adherent (Figure 5e, lower row, left and right panels, arrowheads).

**Figure 6:**
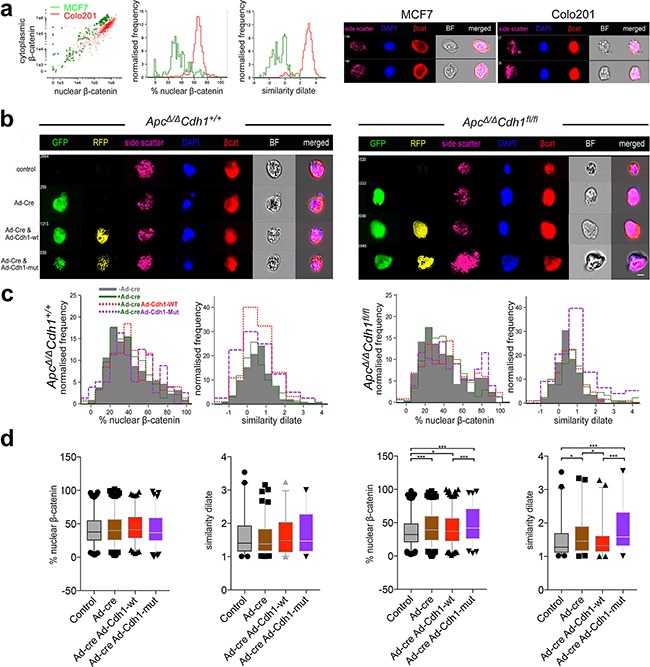
Localization of nuclear β-catenin in *Apc^Δ/Δ^Cdh1^fl/fl^* adenoma organoids following complementation with adenovirus expressing wild-type and EC1 domain mutant of *Cdh1* **a.** Validation of image stream analysis of β-catenin localization in MCF7 and Colo201 cells with cytoplasmic and nuclear β-catenin localization, respectively. Note difference in the % of nuclear β-catenin and similarity dilate between the cell lines, with MCF being cytoplasmic and Colo201 being nuclear. Image stream representative images displaying differential β-catenin localization in the cell lines. **b.** Image stream representative images displaying differential β-catenin localization in the adenoma organoid cells with respect to genotype. **c.** Image stream evaluation for nuclear β-catenin utilises either a DAPI mask (left graph, % nuclear β-catenin) or similarity dilate (right graph, direct comparison of DAPI with β-catenin). Shown are the profiles of nuclear vs. cytoplasmic β-catenin localization for non-Ad-Cre-GFP transfected (grey shading) versus Ad-Cre-GFP (green), with either Ad-Cdh1-WT-RFP (red) or Ad-Cdh1-Mut (purple) co- transfected adenoma organoids. **d.** Quantification (mean and 95% confidence interval with outliers) and statistical analysis of the % nuclear β-catenin and similarity dilate (R12 region, Supplementary Figure S14) with respect to genotype and intervention. Note the rescue of the increase of nuclear β-catenin localization in double Ad-Cre-GFP and Ad-Cdh1-WT-RFP transfected *Apc^Δ/Δ^Cdh1^fl/fl^* cells, and the significant overall increase in nuclear β-catenin localization following Ad-Cdh1-Mut (see also Supplementary Figure S14). *p<0.05, **p<0.01, ***p<0.001, one-way ANOVA, one tailed t-test. Scale bar 8 μm.

As the relatively small proportion of cells doubly transfected precluded additional quantitative assessment of bulk gene expression studies and western blots, we utilized imaging flow cytometry (ImageStream) to attempt to quantify the intra-cellular localization of β-catenin following complementation (Supplementary Figure S14 Figure 6a-d). We first validated differential β-catenin localization using imaging flow cytometry with both MCF7 (cytoplasmic β-catenin) and Colo201 (nuclear β-catenin) control cells, and we were able to quantify relative nuclear and cytoplasmic localization, including example images and the more accurate similarity dilate plots (Figure 6a,b). In *Apc^Δ/Δ^Cdh1^+/+^Lgr5CreER^T2^* control cell suspensions, no significant redistribution of β-catenin appears following Ad-Cre-GFP, Cdh1-WT-RFP and Cdh1-Mut-RFP expression (Figure 6b,c,d, left panels). In *Apc^Δ/Δ^Cdh1^fl/fl^Lgr5CreER^T2^* however, expression of Ad-Cre-GFP resulted in detectable increase in nuclear β-catenin labelling compared to non-Cre expressing controls (Supplementary Figure S14, Figure 6b,c,d, right panels). When Ad-Cdh-WT-RFP was also co-expressed, a statistically significant reduction in the proportion of cells with nuclear β-catenin was observed with the similarity dilate function, compatible with a partial rescue and the fluorescent localization by confocal microscopy (Figure 5b,c,d). For Cdh1-Mut-RFP, the effect appeared reversed, with even higher overall cytoplasmic and nuclear β-catenin labelling in the nucleus in expressing cells, again reflecting the fluorescent imaging (Figure 6b,c,d). All of the quantitative cell data from two separate experiments are shown and statistically analysed for nuclear β-catenin localization using image stream (Figure 6d). Analysis of these data support the function for the cytoplasmic domain of E-cadherin as a negative regulator of nuclear β-catenin, but this only appears optimal in reversing the loss of function of E-cadherin when the EC1 domain dependent adhesion function remains intact (Ad-Cdh-WT-RFP). We interpret these observations in relation to the effectiveness of the sequestration of β-catenin, as this may optimally occur in the stable membrane located adhesion complex. Importantly, Ad-Cre-GFP and Cdh1-Mut-RFP double transfected cells still appeared to retain separate non-adherent cells by confocal imaging, some with rounded and flattened cells, also associated with higher levels of nuclear β-catenin. Using Imagestream, Cdh1-Mut-RFP rescued cells also had much higher nuclear β-catenin (Figure 6d), suggesting that β-catenin binding to the cytoplasmic domain of E-cadherin alone is not sufficient to confer complete reversal of the *Cdh1* loss of function phenotype, further implicating the EC1 binding domain as an important component of the E-cadherin mediated negative regulation of the adhesion and EMT phenotype.

## DISCUSSION

### Cdh1 is essential for embryonic development

E-cadherin is an essential protein, as *Cdh1^−/−^* is known to be embryonic lethal in the mouse because of trophectoderm failure by E2.5-E3.5 [37]. Expression of Cre driven by the promoter of villin (*Vil-Cre*), is active in the visceral endoderm from E9 [28], suggesting that *Cdh1^fl/fl^* recombined using *Vil-Cre* might be expected to bypass the post-implantation developmental defect [29]. The embryonic lethality we observed with *Cdh1^fl/fl^Vil-Cre* was, however, also associated with defects in extra-embryonic development. The alternative *Vil-Cre* transgene (Tg*Vil-cre*-997Gum) that uses a 12.4kb fragment of the *villin* gene promoter, is also active in the intestine from E14.5 but not the yolk sac, and has been previously reported in combination with *Cdh1^fl/fl^* [38]. Here it to resulted in lethality, but in the early post-natal period, meaning that conditional and tissue specific Cre activation was required to evaluate *Cdh1^fl/fl^* in the adult mouse intestine [29, 39].

### Cdh1 and Apc combined to generate additive intestinal phenotypes and β-catenin nuclear localization

When we combined conditional alleles of *Cdh1^fl/fl^* with *Apc^fl/fl^* and tamoxifen-inducible intestinal specific Cre transgene (*Vil-CreER^T2^*), we observed rapid and lethal intestinal phenotypes with marked nuclear localization of β-catenin in intestinal cells [24, 29, 40]. *Cdh1^fl/fl^* recombination alone resulted in lethality due to disrupted intestinal barrier function, with increased crypt cellular proliferation and intact E-cadherin in some cells suggested a regenerative response. In keeping with regeneration, increased expression of the EGF receptor and the EGF ligand amphiregulin (*Areg*) were observed, consistent with crypt myo-fibroblast mediated expression of *Areg*, and the *Areg* requirement in post-irradiation intestinal regeneration [41]. The altered localization of secretory cell lineages, in particular Paneth cells within villi, and the abundance of goblet and entero-endocrine cells, may also have been secondary events to the defects in adherence and regenerative function, as reported previously [40]. These findings are consistent with similar intestinal phenotypes following disruption of other components of the adherens complex, in particular *p120*, *EphB3* and *ADAM10* [9, 42, 43].

Our most striking result was, however, the increased nuclear β-catenin localization that appeared in intestinal cells when disruption of *Apc* and *Cdh1* were combined, and the correlation with increased Wnt target gene expression including *CD44* and *c-Myc.* As *EphB* signalling can also be mediated by *ADAM10* cleavage of E-cadherin, and whilst *EphB2* and *EphB3* are also Wnt target genes, the significantly increased *EphB3* expression in the setting of *Cdh1* loss may have been independent of Wnt signalling [44]. Whilst these genes can be up-regulated in inflammation and regeneration, the direct effects of β-catenin do appear more likely to be the driver in this context [45]. With respect to the observation relating to the paradoxical suppression of *Axin2,* this suggests that the *Axin2* may be reduced due to loss of E-cadherin. It is plausible that this may also be an indirect mechanism via inflammatory and regeneration mediated transcriptional suppression of the *Axin2* promoter. Moreover, in support of this hypothesis, Notch signalling also regulates intestinal secretary cell differentiation and regeneration, and can indirectly suppress *Axin2* expression to modify β-catenin supply [46, 47]. The data obtained in this conditional mouse model also have relevance for intestinal inflammatory disease, as chromosome 16q22, which contains *CDH1*, has been identified as a susceptibility locus for ulcerative colitis in a large genome-wide association study [48], as well as in colorectal cancer (see below) [26, 27]. The lack of association in a subsequent GWAS meta-analysis for Crohn's disease, is countered by a specific risk haplotype that had increased cytoplasmic E-cadherin expression due to a truncated protein, supporting the functional role of E-cadherin as a modifier of human inflammatory intestinal disease [49].

### E-cadherin inhibits nuclear β-catenin localization in the context of Apc dependent adenoma organoids

Surprisingly, conditional generation of intestinal adenoma following tamoxifen administration in *Apc^fl/fl^Cdh1^fl/fl^Lgr5CreER^T2^* did not result in frequent and detectable recombined Cdh1*^Δ/Δ^* alleles, yet subsequent adenoma organoid cell culture manipulated using adenovirus Cre vectors, induced recombination of Cdh1 alleles. The observed EMT phenotype and β-catenin nuclear localization subsequently observed, also strongly support E-cadherin as an inhibitor of β-catenin activated Wnt signalling, mirroring the β-catenin up-regulation in the intestinal crypt-villous units. The cell EMT phenotype was rescued in cells complemented by expression of wild-type E-cadherin, in terms of adhesion and cell shape, but not by an EC1 extra-cellular domain E-cadherin mutant that lacked homophilic binding. These data do not exclude other regulators of EMT, including those related to the activation of β-catenin and compensation for E-cadherin depletion, that may have not been reversed by *Cdh1* complementation. For example, multiple levels of regulation exist to decrease E-cadherin expression such as transcriptional repressors of *Cdh1* mRNA, including slug, snail, twist, and ZEB-1, but these also modify other pathways [50–55]. Activation of these pathways following E-cadherin loss of function may also account for the apparent reduction in proliferation and apoptosis detected in remaining E-cadherin null cell populations 4-6 days after recombination. It therefore remains unknown whether there exists heterogeneity in the cellular response to the loss of E-cadherin, and this partly may be determined by cellular context. In the evaluation of potential EMT pathways in intestinal adenoma and organoids, *Cdh1* was never completely disrupted in *Apc*^Δ/Δ^*Cdh1^fl/fl^Lgr5CreER^T2^*, and so this limited the potential value of a specific transcriptomic analysis to identify these signals [30]. Importantly, these data also suggest that E-cadherin tumor suppressor function may not act through the cytoplasmic domain-β-catenin binding alone, but may also require EC1 domain binding in *trans* and subsequent stable adhesion complex formation. One hypothesis is that cells with complete loss of *Cdh1* may have not survived *in vivo*, as *Cdh1^fl/fl^Vil-CreER^T2^* also showed increased caspase mediated apoptosis and detachment, similar to the reports of E-cadherin loss being involved in the onset of anoikis [56]. Subsequent and on going, experiments will need to carefully evaluate the temporal cellular events, including gene expression, that follow *Cdh1* loss of function *in vitro* in intestinal organoids with *Apc*^Δ/Δ^**, as in this case. Importantly, *Cdh1* cell autonomous function also needs to be evaluated in the context of intact *Apc* using stem cell derived intestinal organoids and conditional alleles, where it is still possible adhesion and EMT phenotypes may also arise in specific contexts.

Other experimental evidence supports our observations for the functional role of E-cadherin as an inhibitor of β-catenin. In *Caenorhabditis elegans*, there appear distinct β-catenin homologues for adhesion and signalling functions [11, 57] and in *Xenopus laevis* embryos, over-expression of cadherins results in reduced dorsal axial structures identical to that seen with depletion of maternal β-catenin [58]. In *Drosophila melanogaster,* either a wild-type E-cadherin (*shotgun*) or a dominant-negative truncated form (lacking the extra-cellular domain) were capable of titrating *armadillo* (β-catenin) between a signalling and adherens junction pools, and over-expression of both constructs resulted in phenotypes similar to either *wingless* or *armadillo* mutants [59]. In mammalian cells, over-expression of E-cadherin can suppress cell proliferation via negative regulation of β-catenin [60], independent of the adhesive function of E-cadherin in some instances [61]. Similarly, siRNA knock-down of E-cadherin can result in increased nuclear β-catenin expression and a β-catenin/Tcf4 TOP-FLASH reporter [62]. In the Rip1Tag2 model of β-islet cell carcinogenesis, however, the transition from adenoma to carcinoma appeared stimulated by dominant-negative E-cadherin expression [63]. Moreover, β-catenin dependent Wnt regulation by E-cadherin was observed in a derived cell line, yet expression of a stable mutant of β-catenin did not promote the same tumour phenotypes [64]. Recently, expression of conditional, mutated and stabilised β-catenin in the mouse failed to generate a significant colonic adenoma phenotype unless combined with a *Cdh1* heterozygote allele, suggesting that the adhesion complex sequestration of mutated β-catenin limited its nuclear translocation [25]. Direct functional effect of *CDH1* germ-line nonsense and missense mutations increases risk of Hereditary Diffuse Gastric Cancer (HDGC), and in some instances to lobular breast cancer [65, 66]. The localization of *CDH1* mutations to both extra-cellular and intra-cellular E-cadherin domains confers invasive properties, and is consistent with a dependency on an intact adhesion complex [67]. These data in combination with that presented in this paper further highlight the importance of the functional potential of the recently rs9929218 polymorphism in colorectal cancer susceptibility and prognosis [26, 27].

In summary, these findings have mechanistic implications for human cancers with co-existing *APC* and *CDH1* loss of function, as the enhanced β-catenin function and invasive phenotypes that arise point to the important tumour suppressor function of both the protein binding functions of intact E-cadherin.

## MATERIALS AND METHODS

### Mice

Animal work was performed under a UK Home Office licence, and approved by University of Oxford ethics committee. The mouse lines were: *Cdh1^+/−^* (C57Bl6J/B6D2), *Apc^Min^* called ‘line1’ [31], backcrossed >10 generations onto C57BL/6J. *Apc^fl/fl^* (*Apc^tm1Tno^*) [68], *Cdh1^fl/fl^* (*Cdh1^tm1Jjon^*) [69], *RosaYFP^fl/fl^* (Gt(ROSA)26Sor^tm1(EYFP)Cos^) [70], *Vil-Cre* (Tg(Vil-cre) and *Vil-CreER^T2^* (Tg(Vil-cre/ERT2)23Syr) [28] and *Lgr5^tm1(cre/ERT2)Cle^* (129P2/OlaHsd) were also backcrossed to C57Bl/6J [14]. *Vil-Cre* is active from embryonic day 10. *Vil-CreER^T2^* is activated with exogenous tamoxifen. Genotyping PCRs were performed as reported, briefly (5'-3', forward-F and reverse-R): *Apc* F GTTCTGTATCATGGAAAGATAGGTGGTC and RCA CTCAAAACGCTTTTGAGGGTTG (GAGTACGGGG TCTCTGTCTCAGTGAA for recombined product), *Cdh1* F CCCCAAACTCGTTGATTGAT and R CCATAC ACTGATAATGTCAGA (CCTGCCATGATTGTCATG GAC for recombined product). Genotype combinations were generated by experimental breeding. Embryos were staged by taking mid-day on the day of seminal plug detection as embryonic day 0.5 (E0.5). Weighed embryos were fixed in 4% neutral buffered formalin. *Cdh1^fl/fl^RosaYFP^fl/fl^* and *Apc^fl/fl^Cdh1^fl/fl^RosaYFP^fl/fl^* were crossed with either *Vil-CreER^T2^* or *Lgr5CreER^T2^* mice, and then inter-crossed to generate *Cdh1^fl/fl^RosaYFP^fl/fl^Vil-CreER^T2^Lgr5-CreER^T2^* and *Apc^fl/fl^Cdh1^fl/fl^, RosaYFP^fl/fl^Vil-CreER^T2^Lgr5-CreER^T2^* animals. *Apc^fl/fl^Cdh1*^+/+^ *RosaYFP^fl/fl^Vil-CreER^T2^*/*Lgr5-CreER^T2^* animals were used as controls. Serial weights were compared using an ANOVA with Bonferroni post-test for each time point. Mendelian ratio analysis utilised the χ^2^ test, and other comparisons between genotypes were made using student's t-test or one-way ANOVA. All p-values were two-sided.

### Conditional cre activation

For *Vil-CreER^T2^* activation (age 8-12 weeks), 40 mg.kg^−1^ tamoxifen in corn oil was administered by intra-peritoneal injection on five sequential days (low dose tamoxifen) or 200 mg.kg^−1^ on two sequential days (high dose tamoxifen). Wild-type controls were also injected to ensure the observed effects were not attributable to the tamoxifen. High dose tamoxifen with *Apc^fl/fl^ Lgr5CreER^T2^+* resulted in adenoma formation between 30 and 80 days. Mice were monitored to a defined humane endpoint (scoring system based on sequential assessments of body condition, weight, respiratory rate, evoked response and tumour/intestinal features) at which time they and matched control animals were sacrificed. Intestinal or splenic samples for RNA extraction were snap frozen or collected in RNAlater® (Ambion). Cre recombinase activation was detected using the *Rosa-YFP* reporter and tissue genotyping for recombined *Apc* and *Cdh1* alleles.

### BrdU and EdU labelling

For BrdU, administration of a single intra-peritoneal injection of 100mg.kg^−1^ (Roche, UK) was either 2 hr or 24 hr before dissection, with ≥2 animals of each genotype for each time point. Immuno-labelling used a nuclease based anti-BrdU labelling and detection kit (Roche, UK) at 4°C in a humidified chamber, followed by incubation with anti-mouse-Ig-fluorescein (dilution 1:10) for 2 hr at 4°C. The cumulative frequency of BrdU positive nuclei counted from the crypt base to the villus tip was quantified for at least 40 crypt-villus units per animal. To assay proliferation *in vitro*, adenoma cultures (see below) were labelled with the thymidine analogue 5-ethynyl-2'-deoxyuridine (10μM, EdU) using Click-iT® EdU Alexa Fluor® 555 (Invitrogen, UK).

### Quantitative RT-PCR

RNA was isolated from intestinal samples using TRI reagent (Applied Biosystems, UK). Contaminant genomic DNA was removed using the TURBO DNA-free kit (Applied Biosystems, UK). cDNA was synthesized from 1.5 μg of total RNA using the High Capacity cDNA Reverse Transcription Kit (Applied Biosystems, UK). Real-time PCR (RT-PCR) analysis was performed with a Rotor Gene Q PCR cycler (Qiagen, UK). Standard curves were performed with serial dilutions of cDNA. Experiments were performed in duplicate and expression levels were normalized to *β-actin* and *Hprt* expression levels using the ΔΔCt method. Reference genes were selected using Genorm^PLUS^ [71]. Primer sequences used to amplify cDNA fragments in Supplementary Methods (Supplementary Table S1).

### Expression microarray

RNA was extracted using an RNeasy kit (Qiagen) from small intestine samples taken from 8-14 week-old mice and stored in RNAlater (Sigma) at −80°C. Samples were obtained 4-5 days after injection of tamoxifen to induce the following conditional genotypes (*Apc^+/+^Cdh1^+/+^, Apc^fl/fl^Cdh1^+/+^, Apc^+/+^Cdh1^fl/fl^* and *Apc^fl/fl^Cdh1^fl/fl^*). RNA was hybridized to Illumina Mouse Whole Genome 6 v2 BeadChips (Illumina, San Diego, CA, Wellcome Trust Centre for Human Genetics, Oxford, UK). Data manipulation and analysis were performed using R3.1.1 (R Development Core Team, 2015), with microarray-specific packages obtained from the Bioconductor repository (Bioconductor 3.0). Raw data were normalized with ‘lumi’ [72] and Significance Analysis of Microarrays [73] was with ‘siggenes’. Mapping of Illumina identifiers to annotation terms was done with ‘biomaRt’ and data from the Ensembl BioMart database (European Bioinformatics Institute, Cambridge, UK). Gene Set Enrichment analysis was with GSEA 2.0.14 (Broad Institute).

### Fluorescence confocal imaging and analysis

Tissue samples were fixed in 4% (v/v) neutral buffered formalin at RT for 24 hr, before dehydration and paraffin embedding. Sections (5μm) were processed by standard techniques. Briefly, slides were de-waxed in xylene, rehydrated followed by antigen de-masking in sodium citrate buffer (10 mM, pH6.0) in a pressure cooker for 2 min at 125°C and 10 min at 85°C. Washed tissue sections (Tris-buffered saline, TBS, pH7.4) were blocked in 10% goat serum/Tween 20^©^ 0.5% (v/v) for 1 hr at RT followed by incubation with primary antibodies; Axin 2 (rabbit, 1:100, ab32197, Abcam, UK); β-catenin (mouse, 1:200, BD610154, BD laboratories, UK); Chromogranin A (rabbit, 1:300, 1773-1, Epitomics, USA); Cleaved Caspase 3 (rabbit, 1:200, #9664, Cell Signaling, UK); c-Myc (rabbit, 1:200, #sc-764, Santa Cruz, USA); E-cadherin (mouse, 1:200, BD610182, BD laboratories, UK); EphB2 (goat, 1:200, #AF467, R&D, UK); GFP (chicken, 1:500, ab13970, Abcam, UK); Cre (mouse, 1:500, # 3120, Millipore, UK); Ki-67 (rabbit, 1:300, RM9106, Thermo Scientific, USA); Lysozyme (rabbit, 1:500, A0099, Dako, Denmark); Mucin 2 (rabbit, 1:500, sc-15334, Santa Cruz, USA) and Villin (mouse, 1:50, sc-58897, Santa Cruz, USA) at 4°C overnight. After 3 washes in TBS tissue sections were incubated with secondary fluorescent-conjugated antibodies (goat anti-mouse or goat anti-rabbit, Alexa-488, −555, −594, or −647, 1:300, Invitrogen, USA, or goat anti-chicken dylight-550, Abcam, UK) for 2 hr at RT or biotinylated antibodies as per manufacturer's instructions (Vectastain elite avidin-biotin complex kit, Vector Laboratories). After washing, fluorescent-labeled tissue sections were counterstained with DAPI (1:5000, D9663, Invitrogen, USA) and mounted with Prolong gold anti-fade reagent (P36930, Invitrogen, USA). Fluorescent images were acquired using a confocal microscope Olympus Fluoview FV1000. Sections labeled using a biotinylated secondary antibody were counterstained with haematoxylin, dehydrated through an alcohol series and mounted with Depex mounting medium (Electron Microscopy Sciences, USA). Images of H&E sections were acquired with an Olympus microscope BX60 with a Nuance CCD system. For adenoma, fixation was in 4% (v/v) paraformaldehyde in PBS 10 mins at RT that also resulted in dissolution of matrigel. Each sample was washed in PBS three times, blocked (1 hr) with 10% serum (same source as secondary antibody, Vector laboratories), 0.5% Triton x100 in TBS (v/v). Samples were incubated with the primary antibody (vimentin (rabbit, 1:200, ab35939, Abcam, UK), twist (rabbit, 1:200, ab49254, Abcam, UK), fibronectin (rabbit, 1:200, ab23750, Abcam, UK) in 3% serum, 0.3% triton and TBS, following 18-24 hr washed in TBS (3x 3 minutes) and incubated with secondary antibody in TBS for 2 hours as above.

The position of Paneth cells (identified by positive lysozyme immuno-labelling) for different genotypes and the distance from the crypt base to a given cell was measured in μm using ImageJ software. Immuno-labelling for mucin 2 and chromogranin A identified goblet cells and enteroendocrine cells, respectively. CC3 labelling was used to identify apoptotic cells. For each marker, positive stained cells per crypt-villus unit were counted to generate an average number of positive cells per crypt-villus unit per animal. At least 3 animals of each genotype and at least 20 crypt-villi units were assessed per animal. Nuclear β-catenin was quantified to assess Wnt pathway activation. Images from at least three animals of each genotype were assessed and for each region (crypt or villus) over 1000 cells were assessed for co-localization of DAPI and nuclear β-catenin. Results were plotted using Graphpad Prism. Heat maps were created using R software [74].

### Western blot analysis

For Western Blot analysis 1x10^6^ cells (MCF-7, HT29, Colo201, Colo320 and HEK293T) were grown. Homogenisation of cells was performed in RIPA buffer (50 mM Tris-HCl pH 8.0, 150 mM NaCl, 1% Triton X-100, 0.5% sodium deoxycholate, 0.1% SDS) in the presence of 2x protease-phosphatase inhibitor cocktail (Halt cocktail, Pierce) at 4°C. After centrifugation at 13,000 rpm at 4°C for 30 min, supernatants, consisting of the cleared lysate, were collected and stored at −80 °C. The concentration of total protein in lysates was determined using the CB-X Protein Assay (G-Biosciences). Volumes of lysate, containing 5 μg protein, were mixed with equal amounts of 2x Laemmli SDS loading buffer (Sigma) and were denatured by heating to 95°C for 5 min. Denatured lysates were centrifuged at 13,000 rpm before being electrophoresed on a 10% SDS polyacrylamide resolving gel (Mini-Protean 3, Bio-Rad). Proteins were transferred to a PVDF membrane (Immobilion P, Millipore) by electroblotting (Mini-Protean 3, Bio-Rad) and membranes were incubated with blocking buffer (5% non-fat milk in TBS/0.1% Tween-20 (TBST) for 1 hour at room temperature. Membranes were incubated (2 hr) with primary antibodies in blocking buffer: E-Cadherin (BD; 1:3,000) and β-Actin (Abcam, 1:20,000). After 3 x 5 minute washes with TBST, membranes were incubated with HRP-conjugated secondary antibodies (Dako, 1:2000) for 1 hr at RT in blocking buffer. Membranes were washed (3 x 5 min) with TBST, signals were developed for using an enhanced chemo-luminescence system (Promega, UK).

### Adenoma culture and adenovirus transfection

Adenoma culture was adapted from [15, 75]. Briefly, caecal (colonic) tumors were utilised due to their larger size, cut into small pieces in sterile PBS (4°C), washed to remove debris and incubated in 5mM EDTA in PBS for 10 min at room temperature, shaken vigorously and washed using PBS (without calcium and magnesium) to remove EDTA. Cells were incubated in Trypsin (0.5mg/ml), supplemented with 100U DNase (at 37°C for 30 minutes) washed in ADF medium (DMEM/F12, Invitrogen, UK) and warmed to 37°C. The cell solution was shaken vigorously, centrifuged at 1200rpm for 3 minutes and the supernatant removed. The remaining adenoma cells were then re-suspended in ADF, dissociated, and pelleted five times, with adenoma cells being passed through a 70μm cell strainer, washed and re-suspended in 1ml ADF, and counted using a NucleoCounter (Chemotech) that assessed cell number, the proportion of single cells and viability. 3000 viable cells in 35μl were combined with 35μl matrigel per well and cultured on glass coverslips in 24-well plates. The plates were incubated at 37°C for 10 minutes to solidify the matrigel, then 500μl of ADF supplemented with N2 (Invitrogen, UK), B27 (Invitrogen, UK), EGF (5ng.ml^−1^, Peprotech) and Noggin (0.1μg.ml^−1^, Peprotech), without R-Spondin. Adenoma culture could be maintained by weekly passage and plating in fresh matrigel. Adenovirus expressing Cre recombinase under the cytomegalovirus (CMV) promoter (Ad-Cre, Vector Biolabs) was used at a multiplicity of infection (MOI) of 500 and transfected on the day of adenoma harvesting culture before being embedded in matrigel. Immuno-labelling with an anti-Cre recombinase antibody as used to detect Cre recombinase expression and anti E-cadherin antibody to detect loss of E-cadherin expression.

As interpretation of immunolabelling results can be subjective, a method was developed to provide an objective assessment of the labelling of individual markers. For each marker, at least 4 adenomas were assessed from each of the *Apc^fl/fl^Cdh1^fl/fl^Lgr5CreER^T2^*Ad-Cre- and *Apc^fl/fl^ Cdh1^fl/fl^Lgr5CreER^T2^*Ad-Cre+ groups. Supplementary Figure S12 outlines the methodology. Using Olympus Fluoview viewer software the adenoma was divided into zones: an outer zone where there was no membrane bound E-cadherin, an inner zone where there was normal appearance of membrane bound E-cadherin, and a transition zone where cells had a mixture of intact and disrupted E-cadherin. Based on distance from the centre of the adenoma, the inner zone was divided into two: a central ‘50%’ area and a surrounding ‘100%’ area. The rationale for the 50/100 division was the 3D structure of the adenomas could results in inconsistent labelling of the central area with some central cores hollow and thus barely labelled, and others strongly labelled. *Apc^fl/fl^ Cdh1^fl/fl^Lgr5CreER^T2^*Ad-Cre- adenomas did not have a transition or outer zone as all cells had intact membrane bound E-cadherin. Once the adenoma was divided into zones, only the channel with the marker of interest was selected and this image was exported to ImageJ where it was converted to grayscale. ImageJ tools were then used to measure the fluorescence of the cells in each area and cells were manually counted to calculate a ‘mean fluorescence per cell’ measurement for each zone. For each marker at least 1000 cells were assessed, but often more (over 7000 in some cases). For each image the mean fluorescence per cell in each zone was normalized to that in the 100% zone. For the Wnt targets β-catenin and Axin2, the location of the marker within the cell was also assessed. Using Olympus Fluoview software, DAPI was colored red and the marker of interest green. When the two were colocalised, the nucleus appeared yellow. For each cell the marker of choice was scored as to whether it was predominantly cytoplasmic, predominantly nuclear or whether there was equal nuclear and cytoplasmic labelling. A cell with predominantly cytoplasmic staining was assigned a value of 0, equal cytoplasmic and nuclear staining obtained a value of 1, and predominant nuclear staining was assigned a value of 2. Thus a total value could be generated for each area that reflected the proportion of cells with nuclear localization of the marker. These data was represented as heatmaps generated using R software. For the average fluorescence per cell heat maps data was underwent a Log2 transformation.

For time-lapse microscopy of adenoma culture was on glass coverslips cultured in humidified imaging chamber at 37°C with a Zeiss LSM510 META confocal microscope and LSM5 software. Images were captured every 2 minutes between 24 and 72 hrs using a 10x objective. Files (.lsm) were loaded into FIJI (http://fiji.sc/) and converted to a stack (.tiff) and saved as an uncompressed .avi movie file with 29 frames per second, and representative frames presented.

### Adenovirus expressing murine E-cadherin

CMV promoter driven adenovirus expressing either murine wild-type (*Cdh1^WT^*) or an EC1 domain mutated cDNA, were combined with an IRES for the fluorescent reporter RFP (Vector Biolabs). The two mutations introduced into the EC1 domain were a tryptophan to alanine (W2A), involved in the strand swapping mechanism, and a mutation of the Ca^2+^ dependent binding site that introduces a bulky tryptophan into the binding site where there is normally a serine residue (S78W). The denoted *Cdh1^W2A, S78Y^* is abbreviated to *Cdh1^Mut^*.

### Flow cytometry validation of E-cadherin binding in MCF7 cells

MCF-7 cells were infected with adenovirus expressing wild-type murine E-cadherin (Ad-Cdh1-WT) or a mutant expressing (Ad-Cdh1-Mut) at an MOI=100, and cell-sorted with a BD-FACSDiva 8.0, 3-4 days after transfection in order to enrich for RFP positive cells. Sorted populations were cultured for a further 2 days, to enhance the yield of *Cdh1* expressing cells, before E-cadherin binding. Cells were detached from the bottom of cell culture flasks with 5 mM EDTA in PBS, re-suspended in DMEM+10% FCS media and grown in 15 ml tubes for another 6 hr at 37 °C_._ Cells were then centrifuged at 1000 rpm, re-suspended in DMEM and transferred to 1.7 ml centrifuge tubes pre-coated with 0.1% BSA. Cells were re-suspended in PBS, with and without His_6_ tag labelled recombinant human Fc-E-cadherin (R&D Systems, 100 ug/ml) or recombinant Fc-E-Cadherin with 5 mM EDTA, and were incubated for (1 hr, 4 °C). Subsequently, cells were washed once with PBS and labeled with a mouse anti-His_6_ tag antibody (1:200, Roche) for 30 min at RT. Cells were then washed and incubated with a secondary goat anti-mouse Alexa405 antibody (1:1000, 30 min, RT). Binding of human recombinant Fc-E-cadherin was quantified by flow cytometry (Cyan ADP analyser, Beckman Coulter).

### Cdh1 complementation in adenomas

*Apc^fl/fl^Cdh1^+/+^Lgr5CreER^T2^* and *Apc^fl/fl^Cdh1^fl/fl^ Lgr5CreER^T2^* adenomas were infected with Ad-Cre-GFP (MOI of 100) alone or in combination with Ad-Cdh1-WT-RFP (MOI of 100) or Ad-Cdh1-Mut-RFP (MOI of 100). In order to increase the infection efficiency, adenoviruses were pre-incubated with adenomas in single cell suspension for 10 min at 37°C. Subsequently, adenomas were seeded in matrigel at a density of 10,000 cells per well (24 well plates) and adenoviruses added to the culture medium for a further 6 days. Culture supernatant was then aspirated from each well and the adenomas were washed with ice-cold PBS. Cells were centrifuged at 1000 rpm and washed twice with PBS at 4°C. Cell pellets were then fixed in 4% (v/v) formaldehyde in PBS (15 min). Cells were blocked and permeabilized in PBS with Triton 0.5% (v/v) and 10% (v/v) goat serum (30 min). Fixed and permeabilized cells were labeled with primary antibodies β-catenin and E-cadherin (BD, 1:300, 1 hr, RT). Labeled and washed cells were then incubated with secondary antibodies, either goat anti-mouse IgG_1_ Alexa405 or goat anti-mouse IgG_2a_ Alexa647 (Invitrogen, 1:500, 30 min). For image stream analysis, *Apc^Δ/Δ^Cdh1^+/+^Lgr5CreER^T2^* and *Apc^Δ/Δ^Cdh1^fl/fl^Lgr5CreER^T2^* adenoma organoid cell suspensions were processed, fixed and labeled as above, except for dilutions of mouse anti-β-catenin (BD, 1:500), and secondary antibody, goat anti-mouse IgG_1_ Alexa647 (Invitrogen, 1:1000). Adenoma cells transfected with adenovirus were analysed with an Amnis Image Stream^X^ Mark II using adapted IDEAS software, include the Dilate mask for similarity, where additional DAPI mask pixels are added.

## SUPPLEMENTARY FIGURES








