# Supplementary material for: Epithelial-mesenchymal transition and nuclear β-catenin induced by conditional intestinal disruption of Cdh1 with Apc is E-cadherin EC1 domain dependent
Source: Oncotarget. 2016 Aug 23;7(43):69883–902. doi: 10.18632/oncotarget.11513 (PMC5342522; doi:10.18632/oncotarget.11513)
Supplement: Supplementary file 1 [file oncotarget-07-69883-s001.pdf]

Epithelial-mesenchymal transition and nuclear β-catenin induced by conditional intestinal disruption of *Cdh1* with *Apc* is E-cadherin EC1 domain dependent

SUPPLEMENTARY FIGURES

a

Timepoint		Genotype *Cdh1*$^{+/+}$	*Cdh1*$^{+/fl}$	*Cdh1*$^{fl/fl}$	Total
E10.5	Observed	4	6	8	18
	Expected	*4.5*	*9*	*4.5*	
E11.5	Observed	2	13	5	20
	Expected	*3.5*	*10*	*6.5*	
E12.5	Observed	4	12	1	17
	Expected	*4.25*	*8.5*	*4.25*	
E15.5+	Observed	8	13	0*	21
	Expected	*5.25*	*10.5*	*6.25*	
P0	Observed	8	39	0***	47
	Expected	*8.5*	*23.5*	*15*	

b

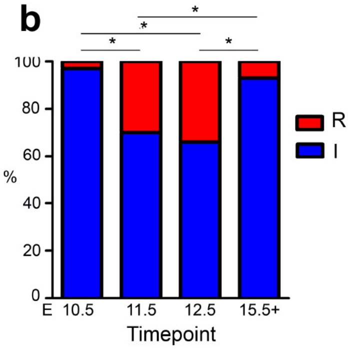

c

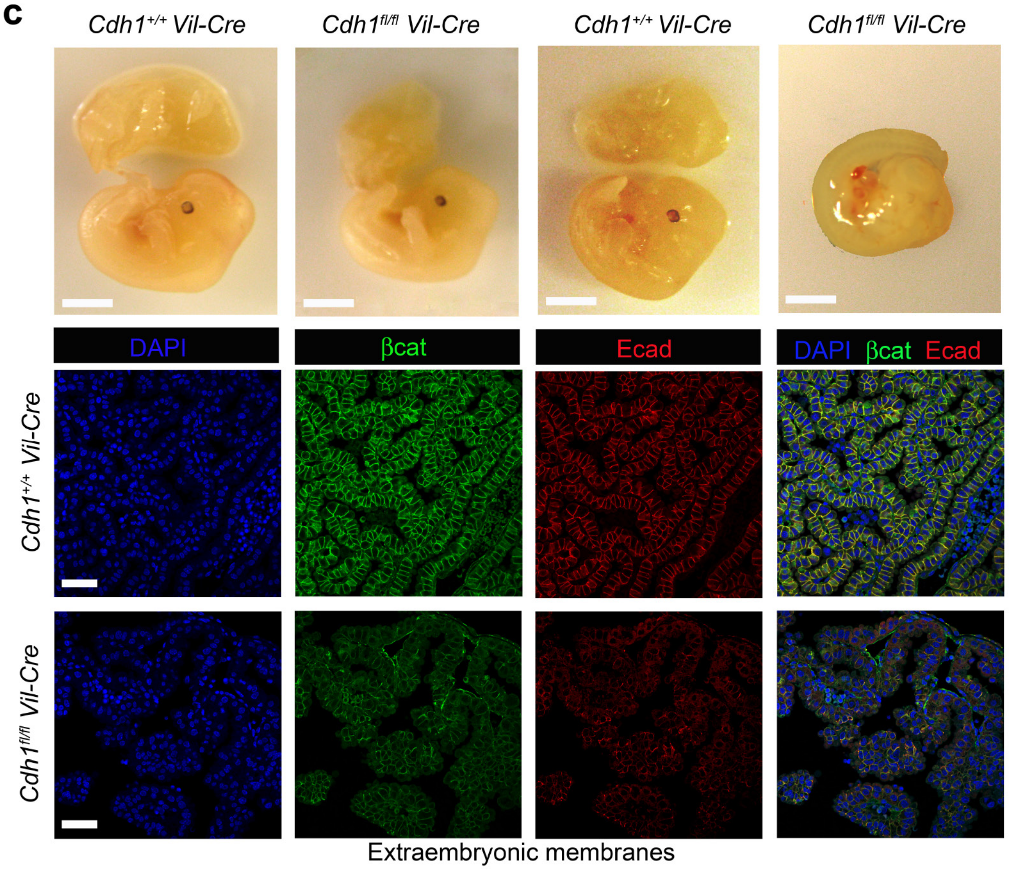

Supplementary Figure S1: The developmental effects of homozygous *Cdh1*$^{fl/fl}$ using *Vil-Cre*. a. Mendelian segregation of embryos and neonates showing loss of *Cdh1*$^{fl/fl}$*Vil-Cre* embyros from E12.5 and significant deviation from expected Mendelian segregation from E15.5. Chi squared test. * p<0.05. *** P<0.001. **b.** Number of embryo resorptions (R) by gestational age for *Cdh1*$^{fl/fl}$*Vil-Cre*. **c.** Top row; examples of embryos at E12.5 of different genotypes, showing smaller *Cdh1*$^{fl/fl}$*Vil-Cre* embryos one of which has disrupted extra-embryonic membranes (right). Scale bars 2 mm. Bottom row; confocal images following antibody labeling of extra-embryonic membranes from the embryos above, with loss of membrane bound E-cadherin and β-catenin with *Cdh1*$^{Δ/Δ}$ homozygosity. Scale bars 50 µm.

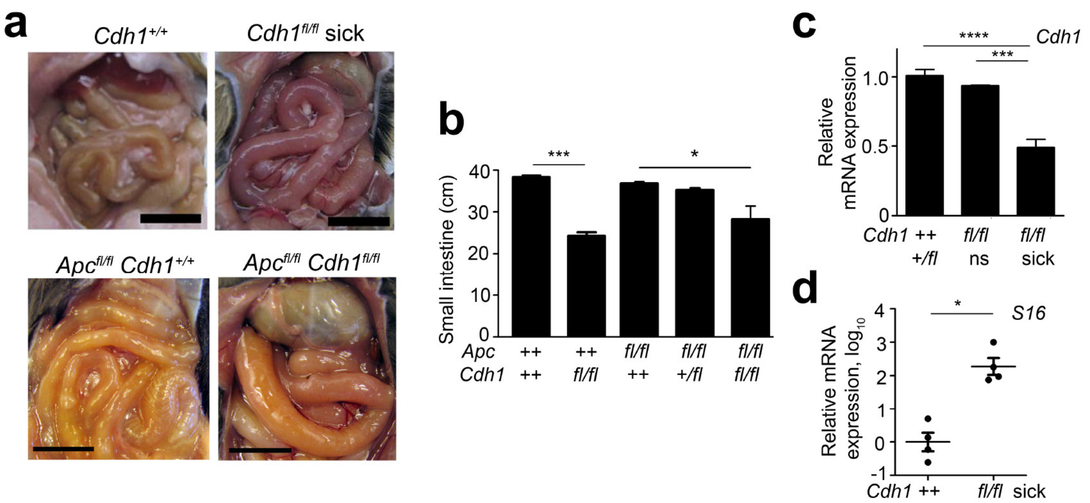

Supplementary Figure S2: *Cdh1* homozygous phenotype with *Vil-CreER*^{T2}. a. Dilated and hyperaemic intestinal appearance in *Cdh1*$^{fl/fl}$ and *Apc*$^{fl/fl}$*Cdh1*$^{fl/fl}$. Scale bar 1 cm. **b.** Small intestinal length is shortened with *Cdh1*$^{fl/fl}$ but not *Apc*$^{fl/fl}$ using *Vil-CreER*^{T2}. Unpaired t- test, ±SEM. **c.** *Cdh1* mRNA expression. *Apc*$^{+/+}$*Cdh1*$^{+/+ \text{ or } +/fl}$ (n=6), *Apc*$^{+/+}$*Cdh1*$^{fl/fl}$ not sick (n=4), *Apc*$^{+/+}$*Cdh1*$^{fl/fl}$ sick (n=4). Unpaired t- test, ±SEM. **d.** Splenic S16 mRNA expression, $\log_{10}$ scale, showing systemic bacteraemia (loss of intestinal barrier function) with *Cdh1*$^{fl/fl}$ homozygosity. Unpaired t-test. ±SEM. * $p<0.05$. ** $p<0.01$. *** $p<0.001$. **** $p<0.0001$.

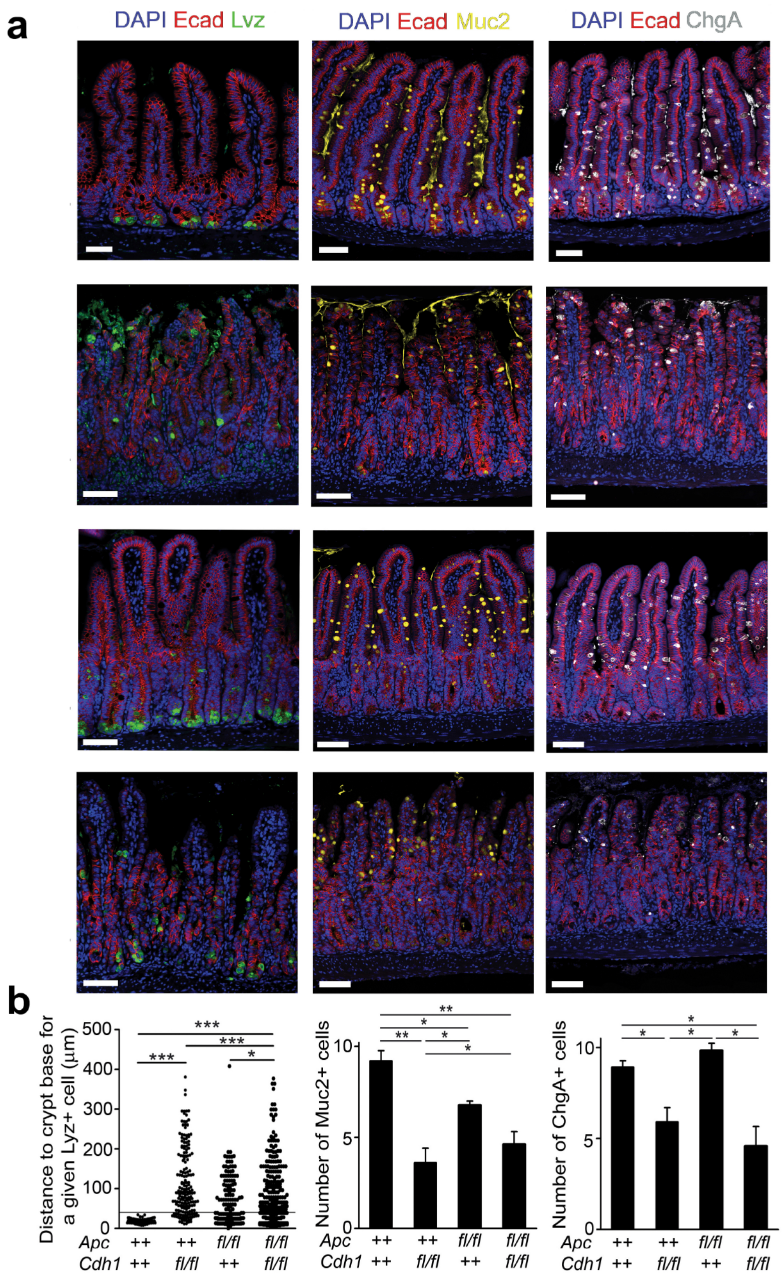

Supplementary Figure S3: The effect of combined *Apc* and *Cdh1* homozygosity on secretory cell lineages. a. Antibody labeling and confocal images for DAPI, E-cadherin and lysozyme (to detect Paneth cells), Mucin 2 (to detect Goblet cells), and Chromogranin A (to detect enteroendocrine cells). **b.** Quantification of the mislocalisation of labelled cells in (a.) by measurement of distance in µm of each cell from the crypt base. Quantification of the average number of goblet or enteroendocrine cells per crypt-villus unit. For each genotype and marker ≥3 mice and ≥20 crypt-villus units assessed per animal. Unpaired t-test for comparison with wild-type samples. Scale bars 100µm.* $p<0.05$. ** $p<0.01$. *** $p<0.001$.

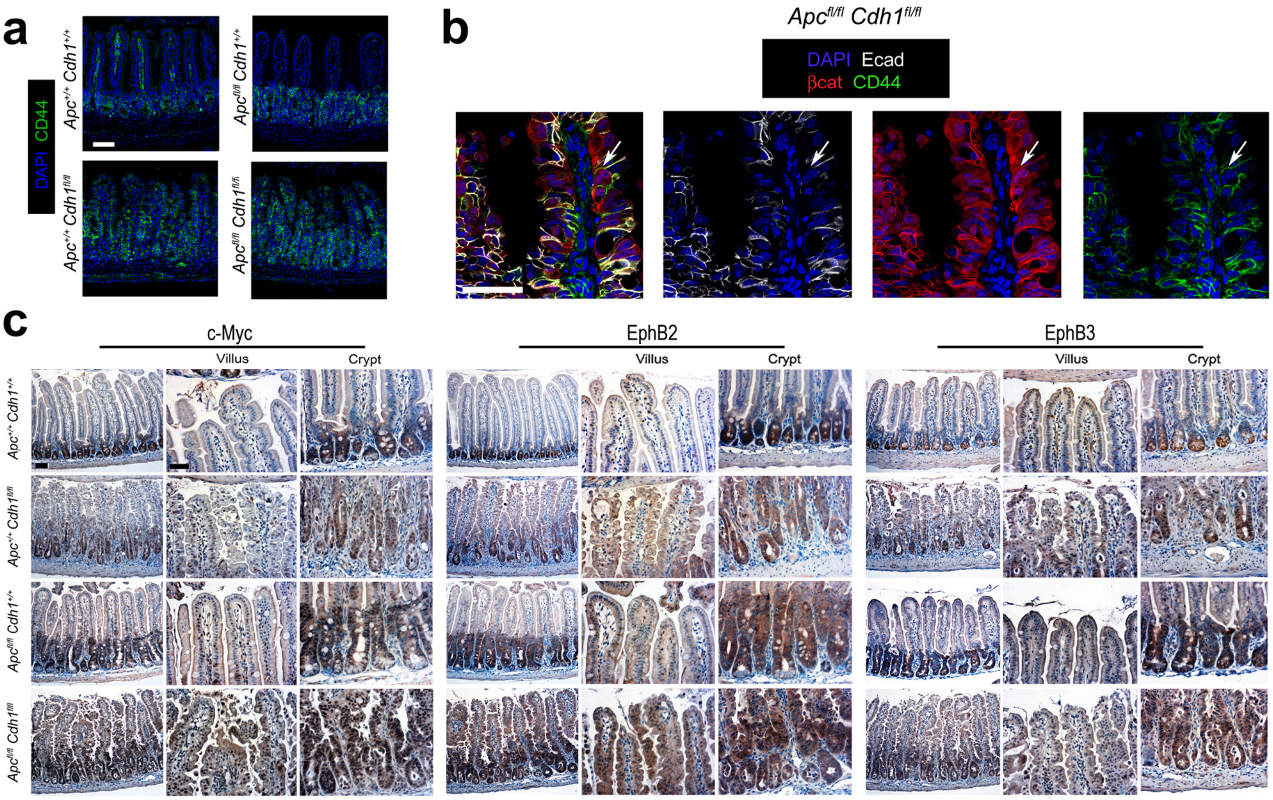

Supplementary Figure S4: The effect of either *Cdh1*$^{fl/fl}$ or *Apc*$^{fl/fl}$*Cdh1*$^{fl/fl}$ homozygosity on Wnt target gene immuno-labeling. a. *Apc*$^{fl/fl}$ dependent fluorescent immuno-labeling for CD44 in the crypt cells. **b.** *Apc*$^{fl/fl}$*Cdh1*$^{fl/fl}$ villous cells lacking E-cadherin and with increased β-catenin and CD44 using fluorescent confocal microscopy. **c.** Expansion of c-myc, EphB2 and to a lesser extend, EphB3 expression beyond the crypt-villous interface in *Apc*$^{fl/fl}$*Cdh1*$^{fl/fl}$ small intestine detected with immuno-cytochemistry. Scale bar 100µm.

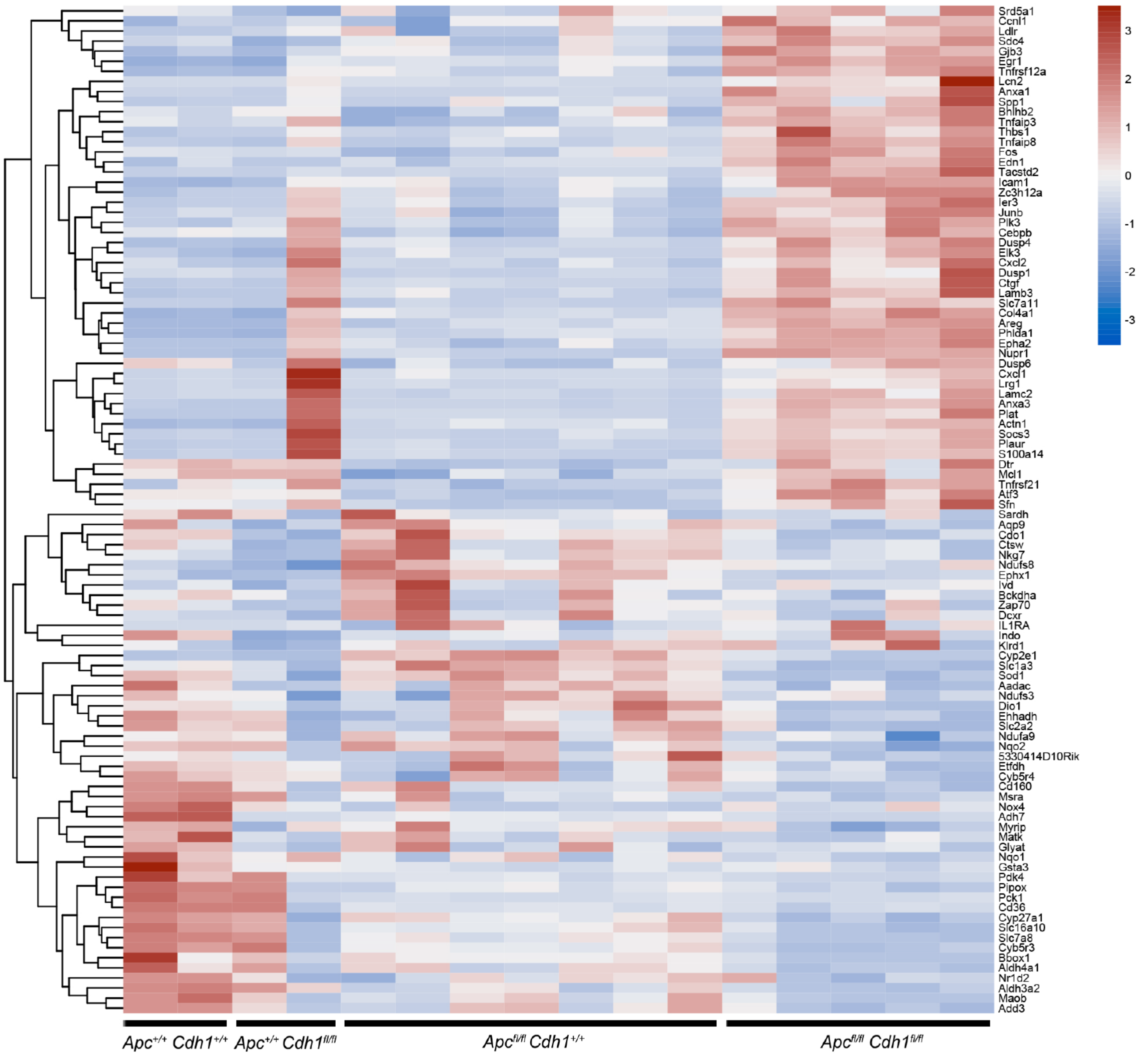

Supplementary Figure S5: Microarray gene expression profiling and gene set enrichment analysis reveals that *$Apc^{fl/fl}$$Cdh1^{fl/fl}$* homozygosity results in up-regulation of *Wnt* target genes, Amphiregulin and inflammasome gene expression signature. Gene Set Enrichment Analysis (GSEA) on microarray data using all of the gene sets in the Molecular Signatures Database v4.0 (Broad Institute). Leading edge sets (LES) from this analysis contained 955 genes, all of the the top 100 genes occurred most frequently in LES (Heatmap). Most enriched was Areg, which encodes amphiregulin and was present in 8 LES. In order to define LES genes, filters were applied to select from these genes; for the most recent Sanger COSMIC Cancer Genes census. (http://cancer.sanger.ac.uk/cancergenome/projects/census/), for the 'inflammasome', Gene Ontology (GO) term annotated as 'inflammatory'. A total of 5 genes were annotated for both cancer and inflammation: Fcgr2b, Egfr, Tnfaip3, Fas and Myd88. (GSE20715_0H_VS_24H_OZONE_TLR4_KO_LUNG_DN, GSE20715_0H_VS_48H_OZONE_TLR4_KO_LUNG_DN, GNF2_SERPINB5, GNF2_CDH3, DELYS_THYROID_CANCER_UP, PEDERSEN_METASTASIS_BY_ERBB2_ISOFORM_1, GSE9988_LOW_LPS_VS_VEHICLE_TREATED_MONOCYTE_UP, GSE14769_UNSTIM_VS_120MIN_LPS_BMDM_DN).

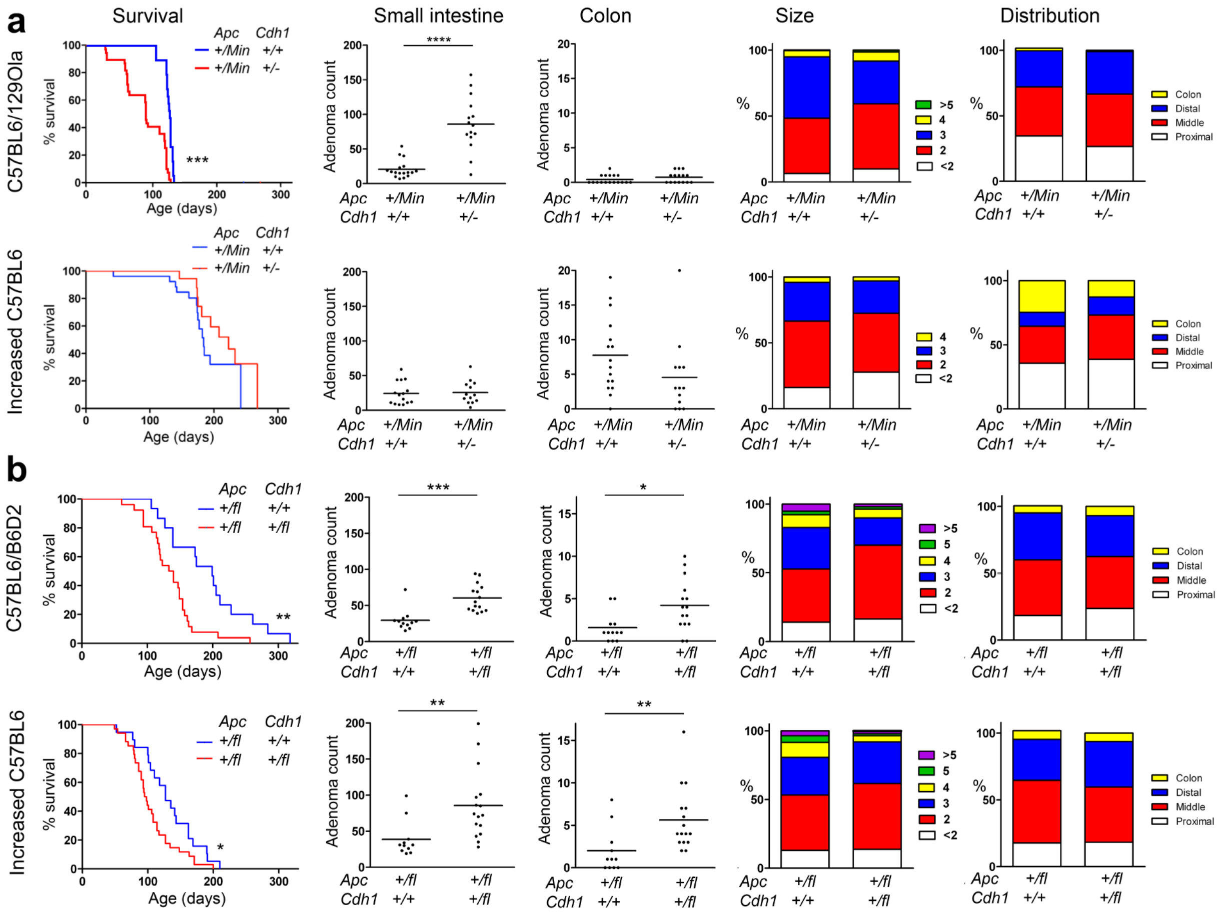

Supplementary Figure S6: Modifier allele effects of *Cdh1* heterozygosity on *Apc* intestinal adenoma on a mixed versus C57BL/6J background. a. $Apc^{Min/+}$ survival and adenoma phenotype on either a mixed C57BL/6J/129Ola (120 days, $Apc^{Min/+}Cdh1^{+/+}$ n=19, $Apc^{Min/+}Cdh1^{+/-}$ n=39, Log-rank test, ***p<0.001) or C57BL/6J background (>10 generations, $Apc^{Min/+}Cdh1^{+/+}$ n=16, $Apc^{Min/+}Cdh1^{+/-}$ n=13, Log-rank test, p=0.1359). **b.** The effect of $Cdh1^{+/fl}$ heterozygosity on $Apc^{+/fl}$*Vil-Cre* survival and adenoma phenotype on either a mixed C57BL/6J/B6D2 ($Apc^{+/fl}Cdh1^{+/+}$ n=15, $Apc^{+/fl}Cdh1^{+/fl}$ n=26, Log-rank test, **p<0.01) or C57BL/6J background (>10 generations, $Apc^{+/fl}Cdh1^{+/+}$ n=19, $Apc^{+/fl}Cdh1^{+/fl}$ n=34, Log-rank test, *p<0.05). Student's t-test to compare adenoma counts between genotypes. Size measured in mm. * p<0.05, ** p<0.01, *** p<0.001.

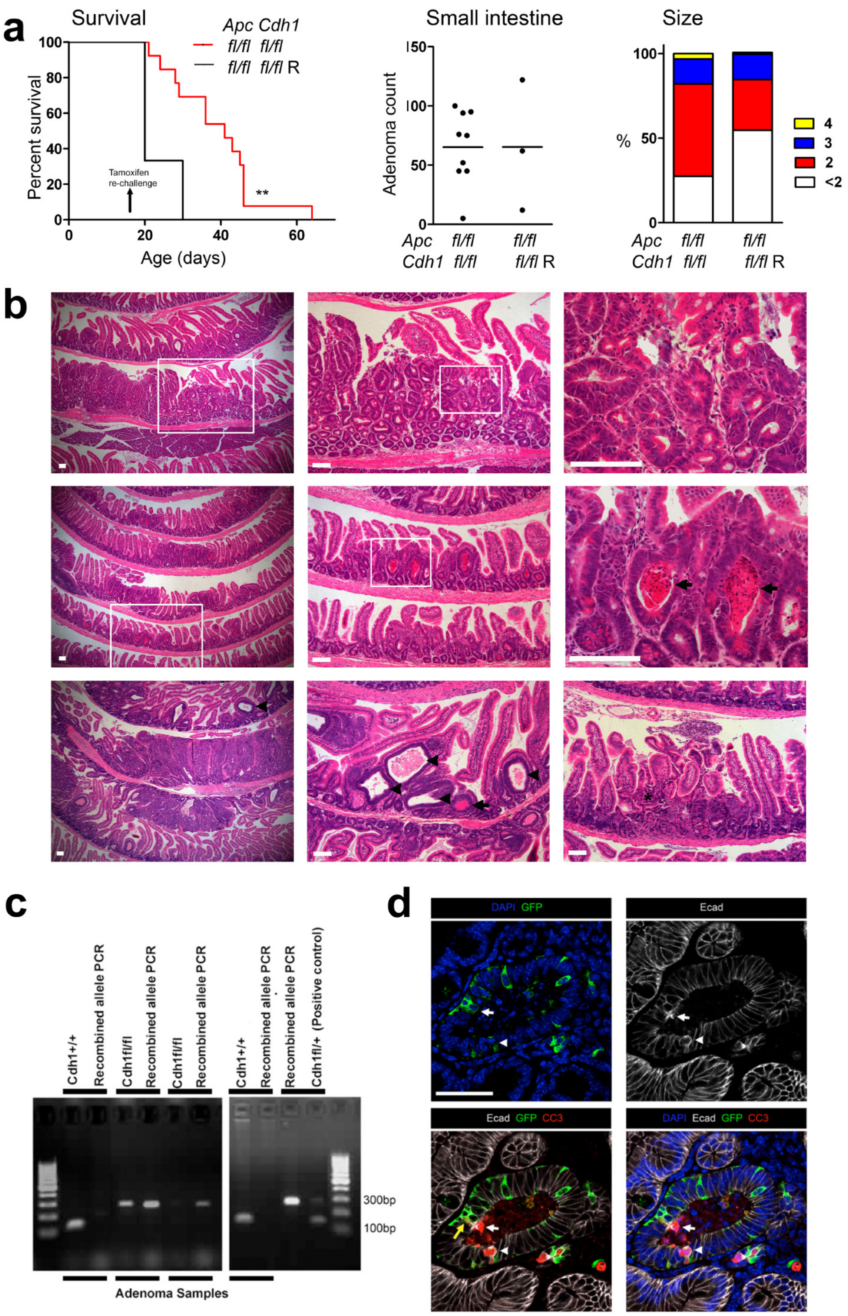

Supplementary Figure S7: *Lgr5-CreER*^{T2} induced *Apc*$^{fl/fl}$ intestinal adenoma select for non-floxed Cdh1 alleles. a. Decreased survival and small intestine adenoma phenotype in *Apc*$^{fl/fl}$*Cdh1*$^{fl/fl}$*Lgr5CreERT2* (n=3) re-challenged (R) with tamoxifen (arrow) (log-rank test). There was a significant difference in adenoma size, with the re-challenged group having a higher proportion of smaller adenomas (student's t-test, p<0.01). **b.** Representative small intestinal histology from *Apc*$^{fl/fl}$*Cdh1*$^{fl/fl}$*Lgr5-CreERT2* re-challenged with tamoxifen. There were no differences in adenoma appearances between genotypes following initial tamoxifen injection (Figure 3a). Top row, widespread adenomatous change, with higher magnification (right). Middle row, adenomatous change with multiple foci of necrosis (black arrows). Lower row, right: large sessile adenomas on a background of diffuse adenomatosis with prominent cystic changes; marked cystic change (black arrowheads) and glands with necrosis (black arrows); disruption of adenoma structure (black star). Scale bars 100μm. **c**. Genotyping PCR of adenomas, including for presence of a recombined *Cdh1* floxed allele (see Methods). Controls: a positive control for recombined *Cdh1* floxed and a control for a known *Cdh1*$^{+/loxp}$ genotype showing the Cdh1+ and Cdh1loxp bands (right). Adenoma samples from*Lgr5-CreER*^{T2}*Apc*$^{fl/fl}$*Cdh1*$^{+/+}$ showing a wild-type *Cdh1* genotyping band, and samples from *Apc*$^{fl/fl}$*Cdh1*$^{fl/fl}$*Lgr5-CreER*^{T2} showing the *Cdh1* genotype in the left lane (very weak in sample 3 indicating a highrecombination rate in this adenoma) and positive recombined allele in the right lane. **d.** Immuno-labeling of small intestine from an *Apc*$^{fl/fl}$*Cdh1*$^{fl/fl}$*Rosa-YFPLgr5-CreER*^{T2} animal for DAPI, E-cadherin, GFP and cleaved caspase 3 (CC3). White arrow indicates *Lgr5* and CC3 positive cells. White arrowhead second *Lgr5* cellalso expressing CC3. Scale bar 50μm.

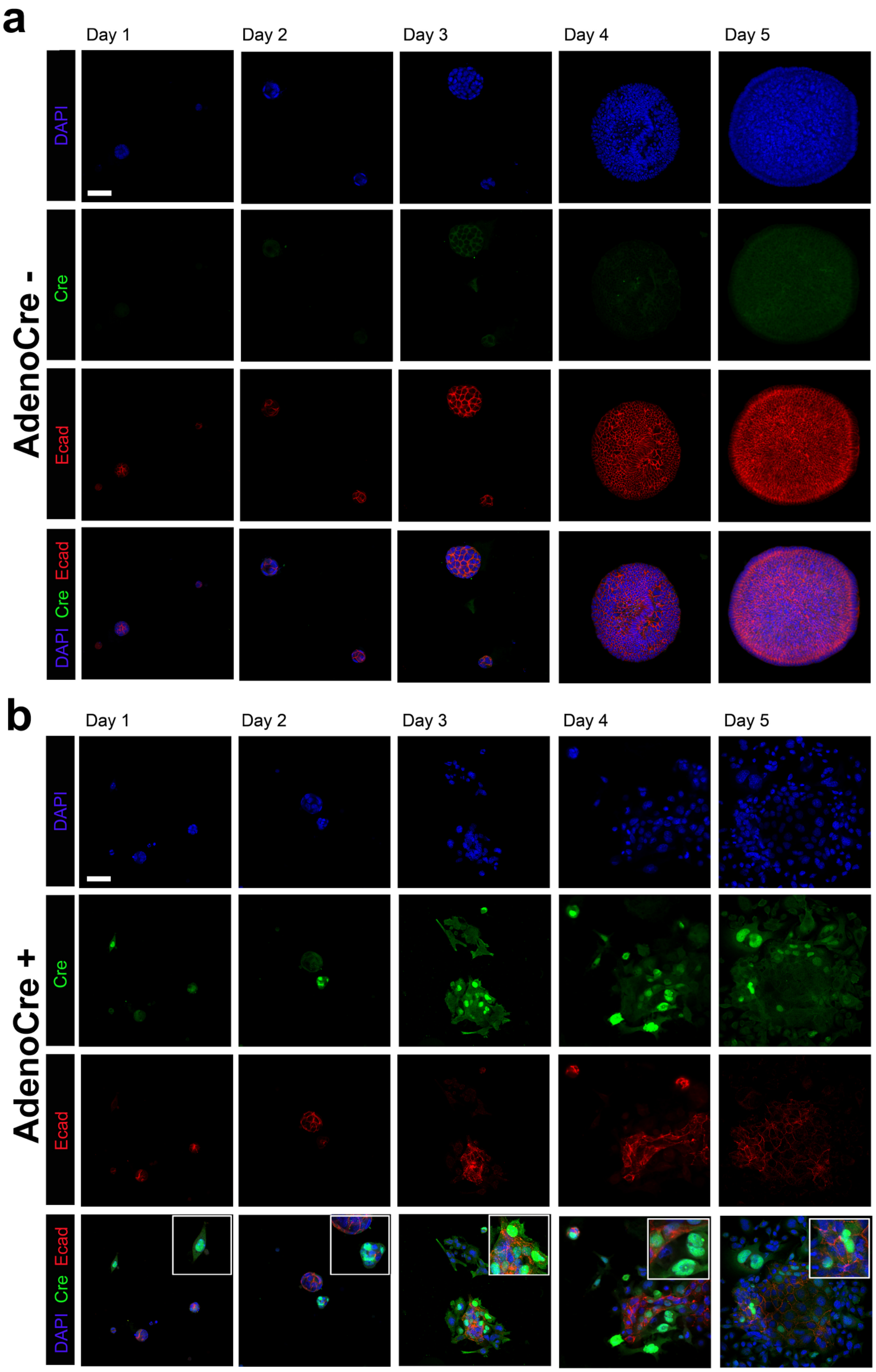

Supplementary Figure S8: Adenovirus Cre induced recombination in *Apc*$^{fl/fl}$*Cdh1*$^{+/+}$ and *Apc*$^{fl/fl}$*Cdh1*$^{fl/fl}$ adenoma organoids. Confocal imaging of *Apc*$^{fl/fl}$*Cdh1*$^{+/+}$ and *Apc*$^{fl/fl}$*Cdh1*$^{fl/fl}$ adenoma organoids over 5 days in culture, with and without Ad-Cre. *Apc*$^{fl/fl}$*Cdh1*$^{fl/fl}$ have disrupted E-cadherin labeling and increased adenoma fragmentation. Inset shows higher power views of Cre recombinase positive cells with disrupted E-cadherin. Scale bar 50μm.

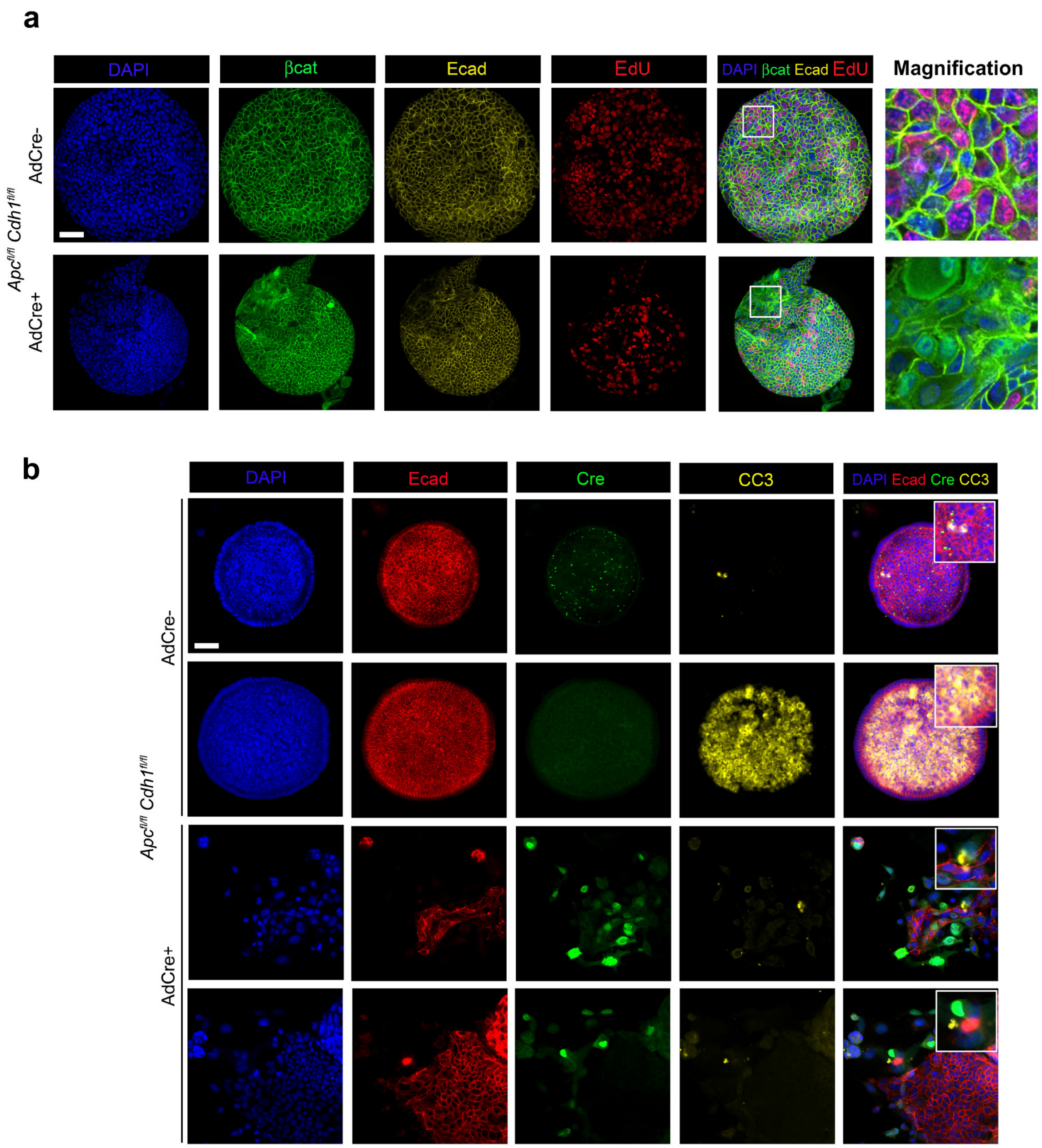

Supplementary Figure S9: Proliferation, apoptosis and epithelial lineage tracing in *Apc*$^{fl/fl}$*Cdh1*$^{fl/fl}$*Lgr5CreER*^{T2} adenoma organoids (days 4-6). a. Cultured adenomas incubated with EdU (2hrs) and then immune-labeled for DAPI, β-catenin, E-cadherin and EdU. In Ad-Cre negative culture, β-catenin and E-cadherin are co-localised at the cell membrane, and EdU positive cells are present in all areas of the adenoma, with some detection of Cre from *Lgr5*. In Ad-Cre positive there appeared fewer EdU positive cells in the area of the adenoma with loss of E-cadherin and increased β-catenin. Scale bar 50µm. **b.** Immuno-labeling with or without Ad-Cre treatment for DAPI, E-cadherin, Cre recombinase and CC3 (cleaved caspase 3). In Ad-Cre negative adenomas, variable CC3 labeling in outer and inner regions and prominent in necrotic cores by day 6. In Ad-Cre positive adenomas, CC3 positive cells were uncommon by day 6. Scale bar 50µm.

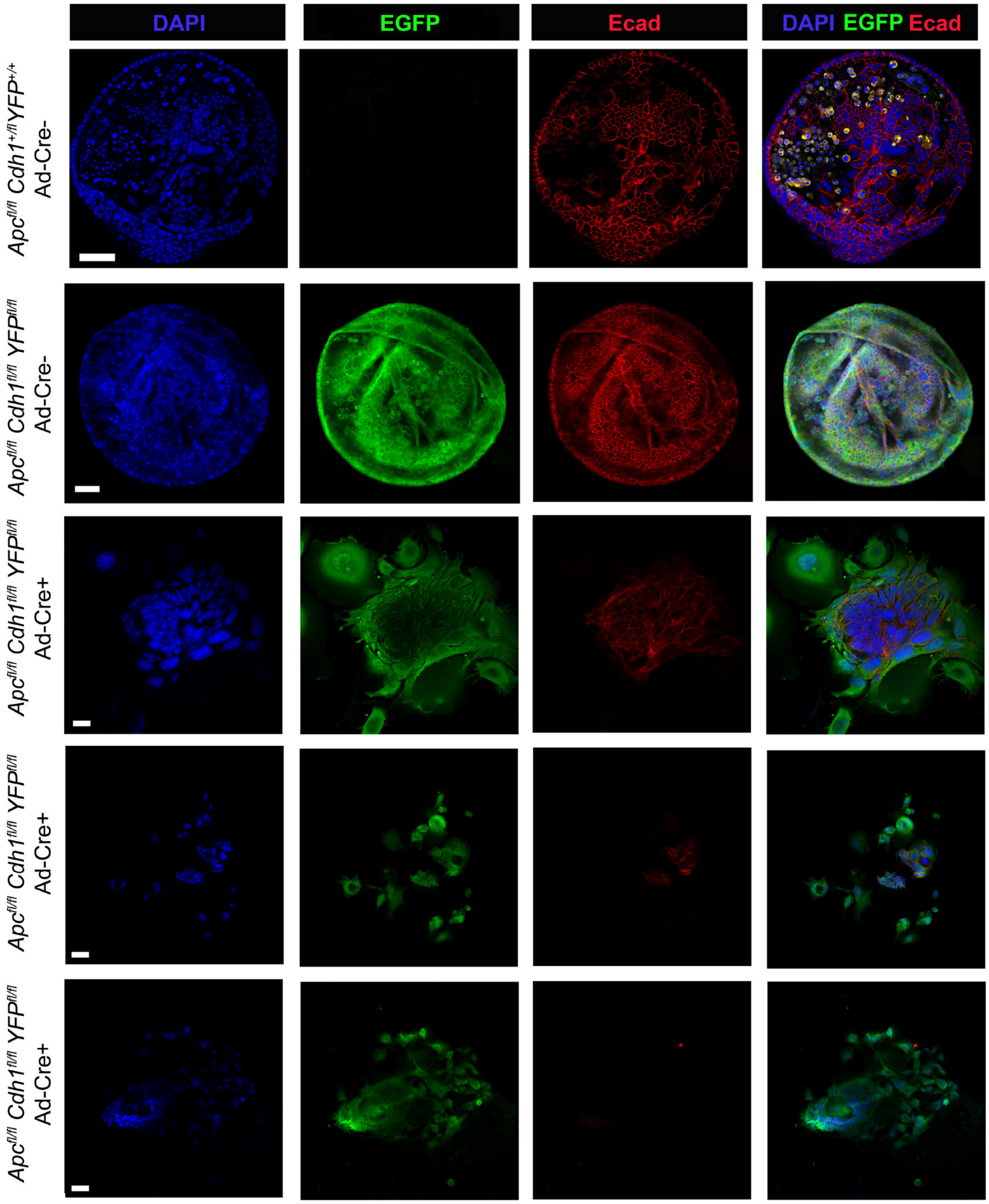

Supplementary Figure S10: Epithelial lineage tracing in *Apc*$^{fl/fl}$*Cdh1*$^{fl/fl}$*Lgr5CreER*^{T2} adenoma organoids using YFP reporter detected with an anti-GFP antibody. Rosa-YFP and EGFP expression detected by immuno-labeling with anti-GFP antibody, DAPI and E-cadherin. Adenoma wild-type for Rosa-YFP, with no YFP expression (Ad-Cre negative), are negative for Lgr5 driven eGFP expression. *Apc*$^{fl/fl}$ *Cdh1*$^{fl/fl}$ *Rosa-YFP*$^{fl/fl}$*Lgr5 CreER*^{T2} Ad-Cre transfected cultures show cells anti-GFP spindle-shaped projections with loss of E-cadherin, confirming these cells originated from either RosaYFP or Adv-Cre-GFP expressing epithelial cells. Scale bars 50µm.

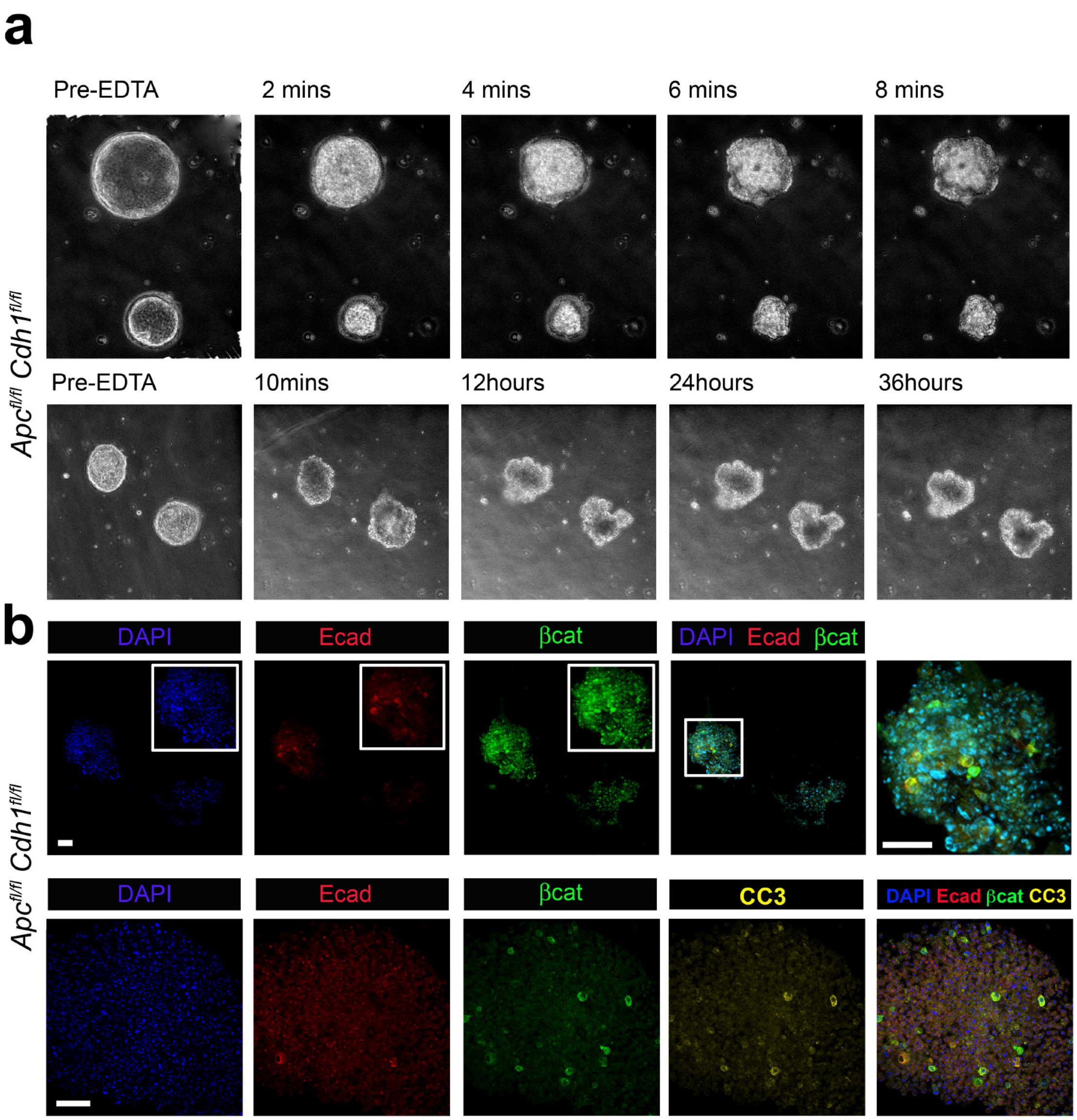

Supplementary Figure S11: The effect of calcium chelation on the phenotype of cultured adenoma organoids. a. Phase contrast images or immune labeling (for DAPI, E-cadherin, β-catenin and cleaved caspase 3 (CC3) ofadenomas treated with EDTA (2.5mM) for 10 minutes followed by calcium free medium for 36 hours. Morphologicalfragmentation occurred over minutes to 1-2 hours (see video). **b**. Adenoma organoids frequently developed pyknotic nuclei, increased β-catenin and associated CC3, but did not reproduce the effects of conditional disruption of *Cdh1* with motile spindle cells. Scale bars 50µm.

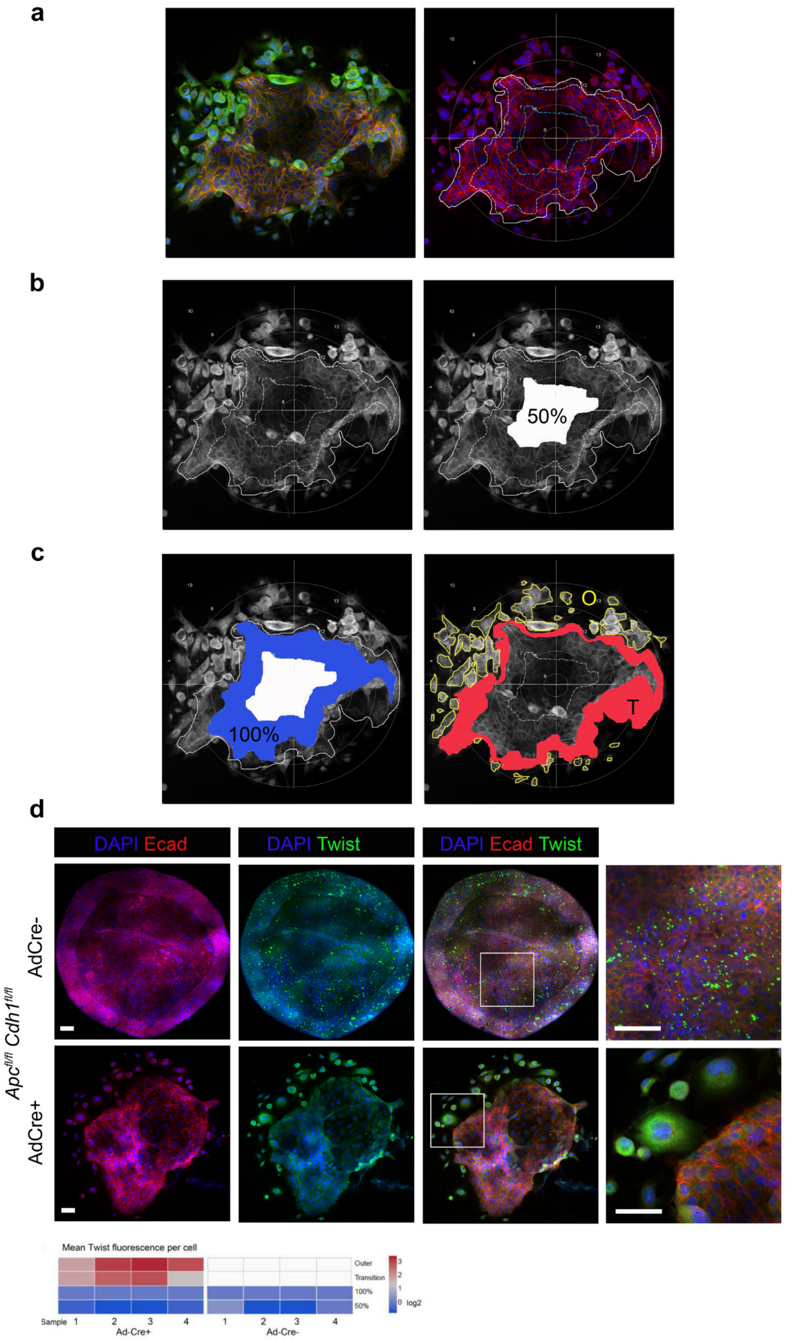

Supplementary Figure S12: Image capture and quantification of adenoma organoids regions for heat map generation. **a**. Image with three channels shown (DAPI in blue, E-cadherin in red, β-catenin in green) used to delineate boundaries between outer and transition zone (based on E-cadherin expression), and '100%' and '50%' zones based on distance from center of the adenoma (see Methods). **b.** Gray scale image of used for fluorescence analysis of marker of interest (β-catenin in this example). **c.**'50%' area marked in white., '100%' area marked in blue. Transition zone ('T') marked in red and cells in outer zone ('O') outlined in yellow. **c.** Example of immune-labeling for DAPI, E-cadherin and Twist showing higher expression of Twist in the outer cells of Ad-Cre cultures lacking E-cadherin. Heat map for mean Twist fluorescence per cell, data log_2 transformed, confirming increased twist expression in Ad-Cre cultures. Scale bars 50μm.

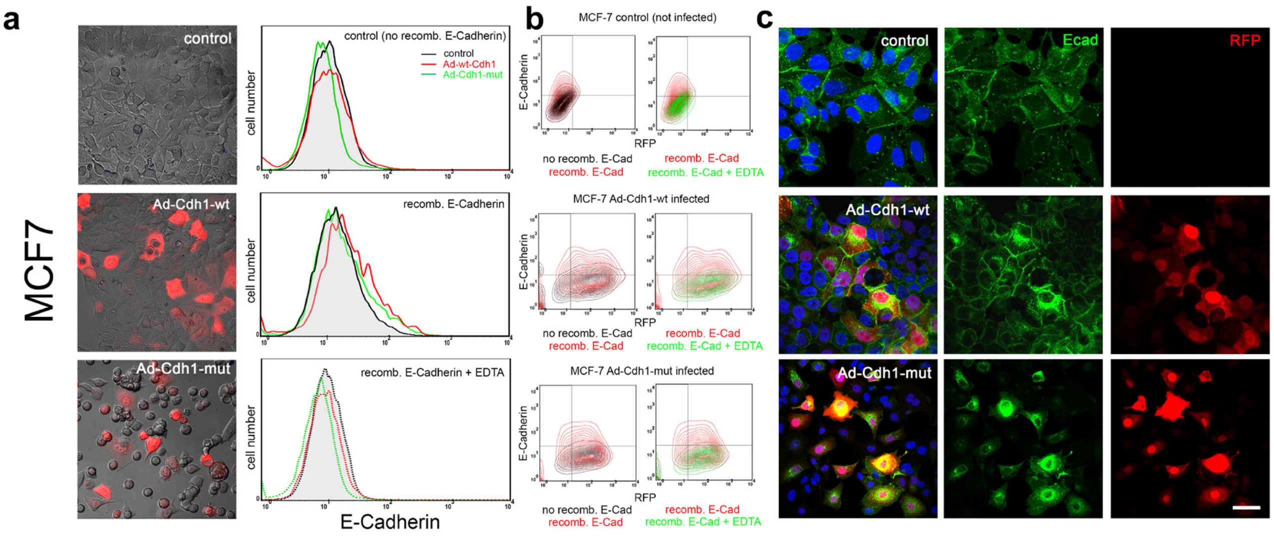

Supplementary Figure S13: Binding of recombinant E-Cadherin to MCF7s transfected with Ad-Cdh1-Wt or Ad-Cdh1-Mut. a. Live cell images show non-transfected (control), Ad-Cdh1-wt-transfected (RFP+ red cells) and Ad-Cdh1-mut-transfected MCF-7s (RFP+ red cells) 3 days post transfection before FACS sorting. FACS plots demonstrate recombinant Fc-E-Cadherin cell binding relative to non-infected control, Ad-Cdh-Wt infected and Ad-Cdh1-Mut transfected MCF-7s. Controls without addition of recombinant Fc-E-cadherin, and combined with EDTA are shown. **b.** Contour plots illustrate increased E-Cadherin detected on the cell surface after binding with recombinant Fc-E-Cadherin, and the decline to baseline after addition of EDTA, in non-infected controls, Ad-Cdh1-Wt-infected and Ad-Cdh-Mut-infected MCF7s. Note, in MCF7 there is detectable endogenous E-cadherin that binds Fc-E-cadherin. When transfected with adenovirus, the shape of the profile changes as RFP is expressed and the plot becomes wider as RFP is on the X-axis. This gives the appearance that Fc-E-cadherin is binding to the mutant Cdh1 protein. Closer inspection of the Ad-Cdh1-wt infected cells however reveals a broader (almost double peak) signal for high E-cadherin labeling, and this reflects the increased expression of wt E-cadherin. **c.** MCF7 cells transfected with mutant E-Cadherin appear to lose cell-cell-contacts and reveal a rounded morphology. This is confirmed by confocal fluorescent images indicating a more cytoplasmic localisation of E-Cadherin accompanied by less localisation at the cell membrane. Scale bars 50 µm.

Mouse Adenoma (transfection)	Genotype	% nuclear β-Catenin (R10)		Similarity dilate R11 = from "0" R12 = from "1"	
		mean	median	Mean (R11/R12)	Median (R11/R12)
714_1	Apc $^{fl/fl}$ & Cdh1 $^{+/+}$	41.95	40.89	0.83/1.66	0.64/1.5
714_2	Apc $^{fl/fl}$ & Cdh1 $^{+/+}$	45.14	43.89	0.79/1.55	0.62/1.35
714_3	Apc $^{fl/fl}$ & Cdh1 $^{+/+}$	43.84	41.39	0.9/1.79	0.67/1.66
714_4	Apc $^{fl/fl}$ & Cdh1 $^{+/+}$	41.36	35.77	0.73/1.7	0.49/1.42
734_1	Apc $^{fl/fl}$ & Cdh1 $^{\mathbf{fl/fl}}$	35.58	31.29	0.69/1.42	0.57/1.26
734_2	Apc $^{fl/fl}$ & Cdh1 $^{\mathbf{fl/fl}}$	40.89 (↑)	37.74	**0.88/ 1.60**	**0.78/1.45**
734_3	Apc $^{fl/fl}$ & Cdh1 $^{\mathbf{fl/fl}}$	38.41	35.04	0.74/1.41	0.65/ 1.32
734_4	Apc $^{fl/fl}$ & Cdh1 $^{\mathbf{fl/fl}}$	46.26 (↑)	40.97	**1.11/1.91**	**0.84/1.81**
743_1	Apc $^{fl/fl}$ & Cdh1 $^{+/+}$	40.83	35.88	0.81/1.55	0.68/1.35
743_2	Apc $^{fl/fl}$ & Cdh1 $^{+/+}$	40.64	37.36	0.80/1.56	0.62/1.4
743_3	Apc $^{fl/fl}$ & Cdh1 $^{+/+}$	44.64	40.88	0.82/1.50	0.63/1.43
743_4	Apc $^{fl/fl}$ & Cdh1 $^{+/+}$	43.46	38.94	1.0/1.69	0.83/1.53
724_1	Apc $^{fl/fl}$ & Cdh1 $^{\mathbf{fl/fl}}$	39.24	34.05	0.75/1.53	0.59/1.31
724_2	Apc $^{fl/fl}$ & Cdh1 $^{\mathbf{fl/fl}}$	46.21 (↑)	42.12	**1.0/1.7**	**0.84/1.51**
724_3	Apc $^{fl/fl}$ & Cdh1 $^{\mathbf{fl/fl}}$	44.66	43.81	0.86/1.56	0.68/1.39
724_4	Apc $^{fl/fl}$ & Cdh1 $^{\mathbf{fl/fl}}$	48.34 (↑)	42.87	**1.15/1.85**	**0.86/1.56**

_1 control
_2 Cre (to knock down E-Cadherin)
_3 (Cre + Ad-Cdh1-wt to rescue with wildtype E-Cadherin)
_4 (Cre + Ad-Cdh1-mut to rescue with mutant E-Cadherin)

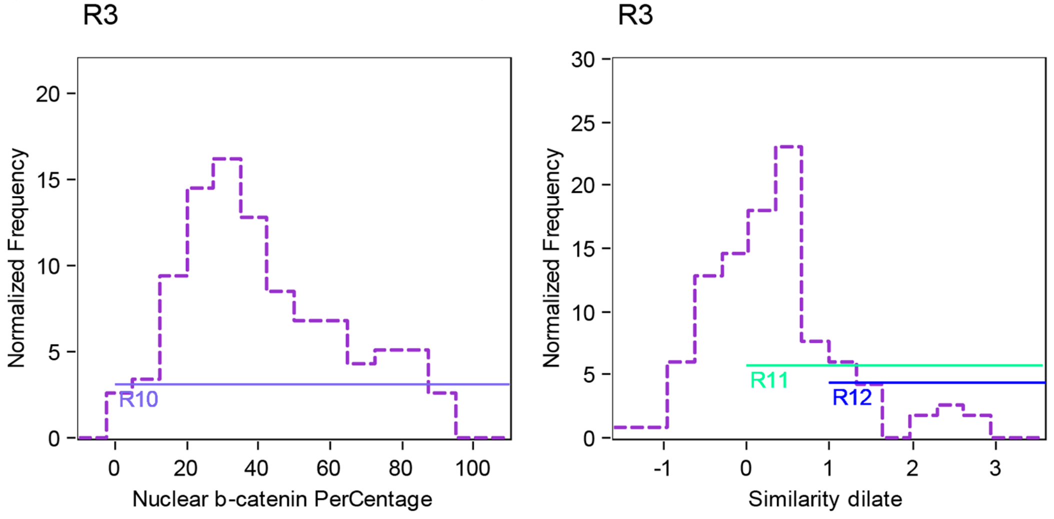

Supplementary Figure S14: Statistical analysis of image stream data. Quantitative image stream data from pooled adenoma from two *Apc*$^{fl/fl}$*Cdh1*$^{+/+}$*Lgr5CreER*^{T2} and two *Apc*$^{fl/fl}$*Cdh1*$^{fl/fl}$*Lgr5CreER*^{T2} mice. Following transfection (conditions listed 1-4), adenoma cell suspensions were labelled and image stream data obtained for β-catenin localisation, and similarity dilate calculated in relation to selection for DAPI nuclear labelling. Floxed *Cdh1* results in increased nuclear β-catenin that is rescued by Ad-Cdh1-WT but not Ad-Cdh1-Mut, inferring EC1 domain dependency.

SUPPLEMENTARY MOVIES

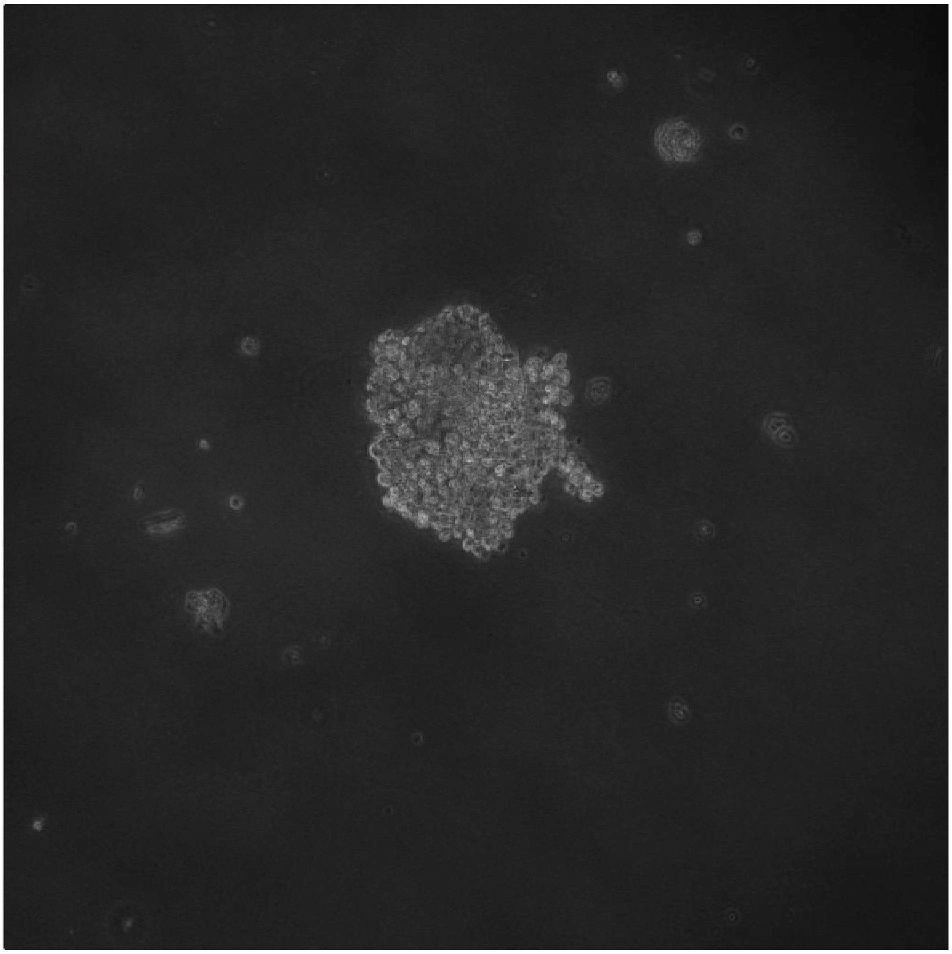

Supplementary Movie S1: EDTA control ApcCdh1flfl.

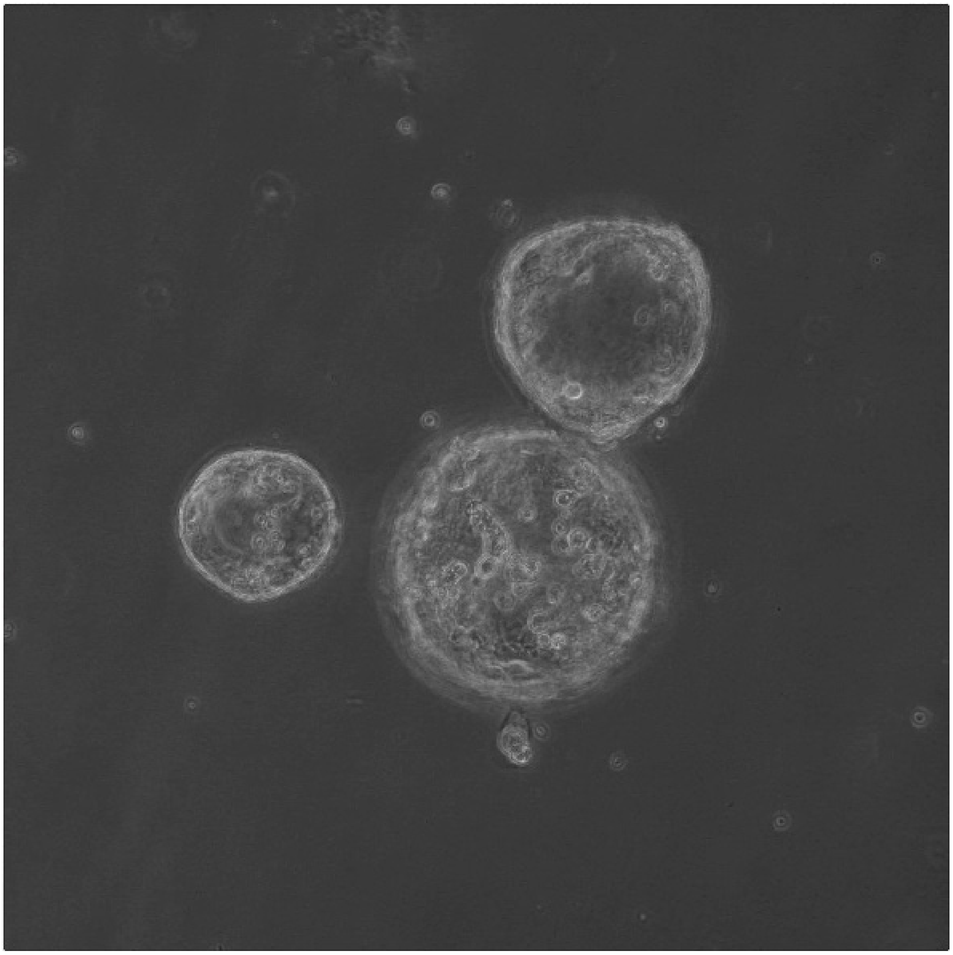

Supplementary Movie S2: Apc_Cdh1_ AdvCreGFP.

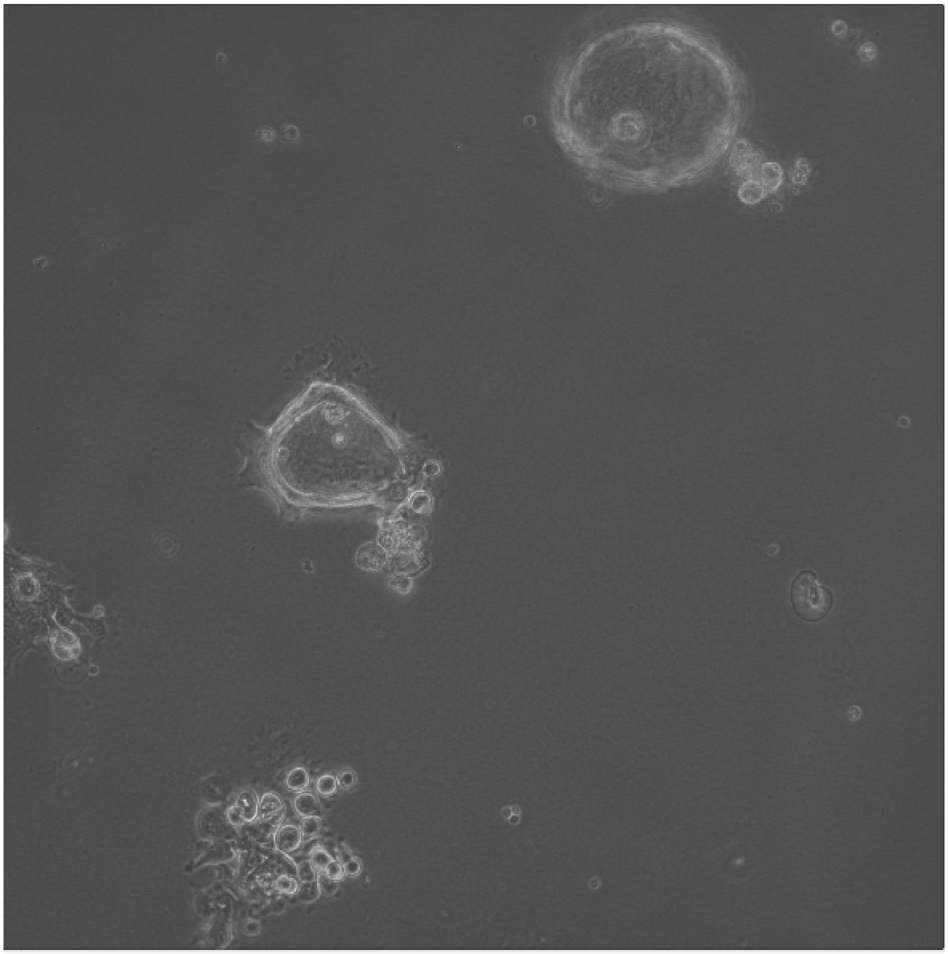

Supplementary Movie S3: Apc_Cdh1flfl Adv-Cre-GFP.
